# Chemical transformation of polyurethane into valuable polymers

**DOI:** 10.1093/nsr/nwae393

**Published:** 2024-12-04

**Authors:** Bo Sun, Jiawei Zou, Weijie Qiu, Shuheng Tian, Maolin Wang, Haoyi Tang, Baotieliang Wang, Shifang Luan, Xiaoyan Tang, Meng Wang, Ding Ma

**Affiliations:** Beijing National Laboratory for Molecular Science, New Cornerstone Science Laboratory, College of Chemistry and Molecular Engineering, Peking University, Beijing 100871, China; State Key Laboratory of Polymer Physics and Chemistry, Changchun Institute of Applied Chemistry, Chinese Academy of Sciences, Changchun 130022, China; Beijing National Laboratory for Molecular Science, New Cornerstone Science Laboratory, College of Chemistry and Molecular Engineering, Peking University, Beijing 100871, China; Key Laboratory of Polymer Chemistry and Physics of Ministry of Education, Center for Soft Matter Science and Engineering, Beijing 100871, China; Beijing National Laboratory for Molecular Science, New Cornerstone Science Laboratory, College of Chemistry and Molecular Engineering, Peking University, Beijing 100871, China; Beijing National Laboratory for Molecular Science, New Cornerstone Science Laboratory, College of Chemistry and Molecular Engineering, Peking University, Beijing 100871, China; Beijing National Laboratory for Molecular Science, New Cornerstone Science Laboratory, College of Chemistry and Molecular Engineering, Peking University, Beijing 100871, China; State Key Laboratory of Polymer Physics and Chemistry, Changchun Institute of Applied Chemistry, Chinese Academy of Sciences, Changchun 130022, China; State Key Laboratory of Polymer Physics and Chemistry, Changchun Institute of Applied Chemistry, Chinese Academy of Sciences, Changchun 130022, China; Beijing National Laboratory for Molecular Science, New Cornerstone Science Laboratory, College of Chemistry and Molecular Engineering, Peking University, Beijing 100871, China; Key Laboratory of Polymer Chemistry and Physics of Ministry of Education, Center for Soft Matter Science and Engineering, Beijing 100871, China; Beijing National Laboratory for Molecular Science, New Cornerstone Science Laboratory, College of Chemistry and Molecular Engineering, Peking University, Beijing 100871, China; Beijing National Laboratory for Molecular Science, New Cornerstone Science Laboratory, College of Chemistry and Molecular Engineering, Peking University, Beijing 100871, China

**Keywords:** polyurethanes, catalytic upcycling, plastic wastes, hydrogenation

## Abstract

Polyurethanes are an important class of synthetic polymers, widely used in a variety of applications ranging from everyday items to advanced tools in societal infrastructure. Their inherent cross-linked structure imparts exceptional durability and flexibility, yet this also complicates their degradation and recycling. Here we report a heterogeneous catalytic process that combines methanolysis and hydrogenation with a CO_2_/H_2_ reaction medium, effectively breaking down PU waste consisting of urethane and ester bonds into valuable intermediates like aromatic diamines and lactones. These intermediates are then converted into functional polymers: polyimide (PI), noted for its exceptional thermal and electrical insulation, and polylactone (P(BL-*co*-CL)), a biodegradable alternative to traditional plastics. Both polymers exhibit enhanced performance compared to existing commercial products. This approach not only contributes to the valorization of plastic waste but also opens new avenues for the creation of high-performance materials.

## Teaser

This study presents an efficient approach for the upcycling of polyurethane waste, utilizing a novel catalytic process that transforms polyurethane into important chemicals and then valuable polymers.

## INTRODUCTION

Plastics, predominantly derived from fossil fuels, have undeniably become a fundamental component of contemporary society [1]. However, the accumulation of end-of-life plastics in the environment is causing a profound ecological crisis worldwide [2]. In the pursuit of a circular economy, plastic waste is increasingly recognized as a valuable carbon resource that can be reintegrated into the chemical/material industry [3,4]. Consequently, devising efficient methods for chemically transforming plastic waste into original precursors or a variety of functional and high-value chemicals is of paramount importance [5,6]. Notably, chemical upcycling, which differs from traditional recycling techniques focused on monomer recovery, presents an advantageous route for enhancing plastic waste management by converting waste into value-added chemicals [7–10]. For example, polyolefins, known for their stability, are difficult to be transformed, but also versatile in their transformation capabilities. They can be converted into various mixture compounds such as mixture of alkanes [11], olefins [12], aromatics [13], or oxygenates [14]. Notably, an elegant and innovative method has been recently reported which allows polyethylene (PE) to react with ethylene, producing a highly valuable single chemical, propene [15,16]. Similarly, there has been substantial progress in developing new methods for the catalytic upcycling of different types of plastic wastes, as evidenced by various studies and advancements in the field [11–22].

Polyurethane (PU), containing urethane bonds and accounting for ∼6% of all plastic waste [23], is typically synthesized from isocyanates and polyols and comes in various forms, including foams, adhesives, and elastomers, depending on the monomers and additives used [24]. Although PU can be relatively easily segregated from waste streams, the complexity of its monomers and its robust cross-linked structure make recycling or upcycling challenging. Techniques such as pyrolysis, hydrolysis, alcoholysis, aminolysis, acidolysis, and glycolysis have been explored for PU recycling [25–31], with methanolysis being a straightforward approach for depolymerization [32,33]. Yet, the resulting low-value polyols and non-virgin monomers (methyl carbamates) from the cleavage of the –C(=O)–O– in urethane bonds make this method less attractive for further PU reproduction. An alternative, more effective method is catalytic hydrogenation, which breaks both –NH–C(=O)– and –C(=O)–O– bonds in an atom-efficient manner to recover basic monomers or derivatives. Several transformation systems have been successfully developed based on homogeneous catalytic hydrogenation [34–37], although their low thermal stability and the challenges in separating catalysts for reuse from reaction systems may limit their practical applications.

We suggest that an effective upcycling strategy for PU should incorporate a carefully engineered heterogeneous catalytic system, which integrates methanolysis with hydrogenation processes. In this context, our proposed strategy involves using CO_2_/H_2_ as the reaction medium along with a heterogeneous catalyst. This catalyst is uniquely designed to facilitate both the conversion of CO_2_ to methanol and the hydrogenation of plastic depolymerization reaction intermediates. This comprehensive approach is anticipated not only to improve the depolymerization efficiency of PU waste and the utilization of CO_2_ waste, but also enable a complete recovery of the designated components. Importantly, we aim to transform these components into valuable materials for the valorization of plastic waste.

**Figure 1.**
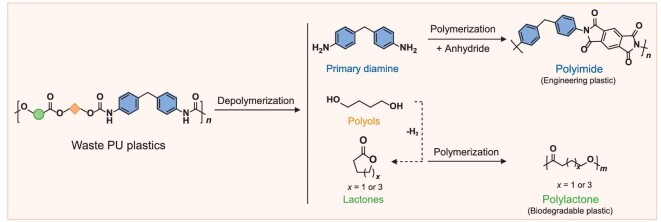
Catalytic upcycling of polyurethane into valuable polymers.

Here we introduce a novel two-step reaction construct to convert polyurethane (PU), containing urethane and ester bonds, into two valuable polymers: polyimide (PI, an engineering plastic) and polylactone (P(BL-*co*-CL), a biodegradable plastic), as illustrated in Fig. 1. During the initial heterogeneous depolymerization step of PU, we employed a mixture of CO_2_/H_2_, a highly effective combination for the catalytic hydrogenative depolymerization of PU into diamines, diols, and lactones. This process achieved a total product yield of 86% using an inverse ZnO-ZrO_2_/Cu catalyst at 200°C. Subsequently, the produced 1,4-butanediol (BDO) was further converted into γ-butyrolactone (BL) using the same catalyst at 220°C. In the subsequent step, the obtained diamine and lactones were utilized to synthesize PI and P(BL-*co*-CL), respectively. Remarkably, from 5 g of waste tyre material, predominantly composed of PU, we successfully produced ∼2.2 g of PI films. These films demonstrated excellent energy-storage capabilities, functioning as dielectric capacitors with a discharge energy density (*U_e_*) of 6.0 J cm^−3^ at 150°C. Concurrently, we also generated ∼0.44 g of polylactone, exhibiting both satisfactory chemical recyclability and ductile properties. This innovative approach not only paves a new path for upcycling PU waste into a range of valuable and functional polymers but also contributes to the realization of a sustainable future.

## RESULTS AND DISCUSSION

First, we synthesized an inverse ZnO-ZrO_2_/Cu catalyst using a method similar to a previously reported procedure [38], with the characterizations presented in Figs S1 and S2 (Supplementary data, XRD and N_2_ physisorption analysis). This catalyst was selected for testing the hydrogenative depolymerization of PU in a CO_2_/H_2_ environment, motivated by the known efficacy of inverse Cu-based catalysts in converting CO_2_ to methanol and hydrogenating polyesters [38,39].

**Figure 2.**
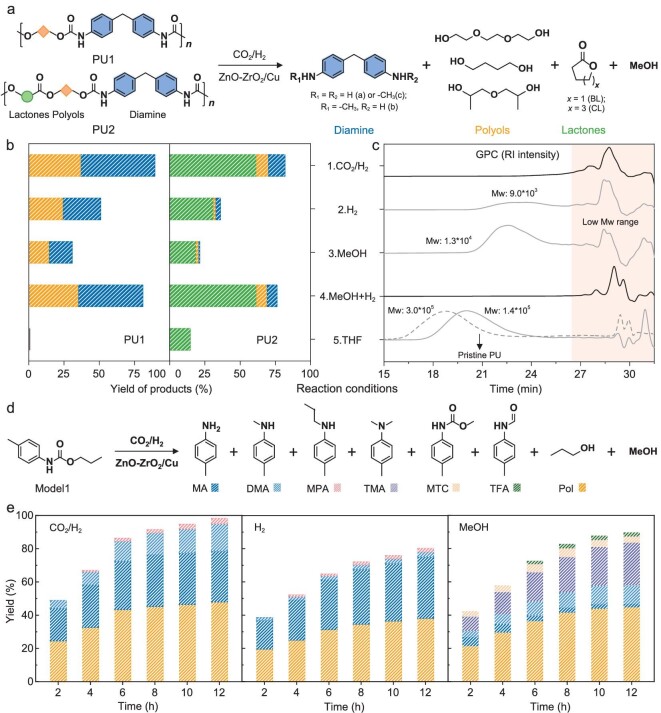
Catalytic hydrogenative depolymerization of PUs and Model1 under CO_2_/H_2_. (a) Schematic reaction pathways for catalytic hydrogenation of PU1 or PU2 over ZnO-ZrO_2_/Cu catalyst under CO_2_/H_2_ (1/3, v/v). (b) Yield of products from catalytic hydrogenation of PU1 or PU2 in different reaction conditions. Conditions: PU1 or PU2 (200 mg), ZnO-ZrO_2_/Cu (200 mg), CO_2_/H_2_ (1/3, v/v, 3 MPa) or H_2_ (2.2 MPa), MeOH (80 µL or none), THF (30 mL), 200°C. (c) Gel permeation chromatography (GPC) measurements of PU2 in different reaction conditions as noted in b. (d) Schematic reaction pathways for catalytic hydrogenation of the Model1 over ZnO-ZrO_2_/Cu. (e) Yield of products from catalytic hydrogenation of Model1 in different reaction conditions. Conditions: Model1 (200 mg), ZnO-ZrO_2_/Cu (200 mg), CO_2_/H_2_ (1/3, v/v, 3 MPa) or H_2_ (2.2 MPa), MeOH (3 mL or none), THF (30 mL), 200°C. The yields of products in (b) were calculated from dividing the obtained mass of products by the input amount of PU. The increased mass ratio of all products from CO_2_/H_2_ was <3.5%. The yields of products in (e) were calculated from dividing the obtained mol of products by the total input mol of aromatic and alcohol segments in Model1.

We then evaluated this catalyst for degrading two types of polyurethane: a synthetic PU1, composed of urethane bonds created from the reaction of 4,4'-methylenedianiline with triethylene glycol (TEG), and a commercial PU2, containing both urethane and ester bonds (Figs S3 and S4). These were chosen as model feedstocks, with PU1 representing a simpler urethane-only structure and PU2 being more representative of real-world plastics. Remarkably, both PU1 and PU2 were completely converted within 4 hours at 200°C under CO_2_/H_2_ conditions (1/3, v/v, 3 MPa) over inverse ZnO-ZrO_2_/Cu catalyst, leaving no residual PUs as confirmed by Fig. 2a, b, and Figs S3–S6. The catalytic depolymerization yielded three types of products: aromatic amines (4,4′-methylenedianiline (**a**); 4-(4-aminobenzyl)-*N*-methylaniline (**b**); 4,4′-methylenebis(*N*-methylaniline) (**c**)), diols (TEG from PU1; BDO and dipropylene glycol (DPG) from PU2), and lactones (BL and ε-caprolactone (CL) from PU2), as shown in Fig. 2a. The product yields were impressive, at 89% for PU1 and 82% for PU2 under condition 1 (Fig. 2b). Additionally, methanol formation was observed, arising from the CO_2_ hydrogenation reaction (Figs S3–S6). This indicates that an effective heterogeneous catalytic system for the degradation of polyurethanes has been successfully established. The mass balance of the catalytic process was calculated through dividing the mass of obtained products related to monomers by the total mass input of polyurethane. Although the calculation does not exclude the involed mass of hydrogen and methyl group from CO_2_, the mass balance is still appropriate to evaluate the efficiency of recovered products from depolymerization of polyurethane. The lower yield of obtained products from depolymerization of polyurethane at higher temperature may be due to the formation of *N*-alkylated byproducts between diamines and diols.

We envision that CO_2_ plays a crucial role in generating methanol, which in turn accelerates the hydrogenative depolymerization of PU. To test this hypothesis, we conducted depolymerization studies under various reaction conditions (Fig. 2b). Initially, we assessed the catalytic depolymerization performance using either hydrogen (2.2 MPa) or a stoichiometric amount of methanol (2 mmol) alone (Fig. 2b, conditions 2 and 3). In both scenarios, we observed a significant reduction in product yields, underscoring the efficacy of the CO_2_/H_2_ combination for efficient catalytic degradation. Notably, the yields in hydrogen (PU1: 51%; PU2: 36%) were substantially higher compared to those in methanol (PU1: 31%; PU2: 21%), suggesting hydrogen's primary role in the hydrogenative deconstruction of PU.

Subsequently, we conducted a control reaction with both hydrogen (2.2 MPa) and a stoichiometric amount of methanol (2 mmol) (Fig. 2b, condition 4). While the activities were comparable, the product yields (PU1: 81%; PU2: 76%) were slightly lower than under condition 1. However, it confirmed the pivotal role of methanol generated *in-situ* from CO_2_ in the depolymerization process. Moreover, in experiments using only hydrogen (Fig. S7) or tetrahydrofuran (THF) solvent without the catalyst (Fig. 2b, condition 5), only lactone products were obtained from PU2. This indicates that hydrogenative depolymerization predominantly occurs over the ZnO-ZrO_2_/Cu catalyst and that the ester bond in PU2 can be partially broken down in THF solvent. To further discriminate the catalytic active sites in the reduction of CO_2_ to methanol, the methanolysis and hydrogenolysis reactions of PU, we carefully investigated the catalytic processes by changing different reaction conditions (Fig. S6). In the CO_2_ reduction process, Cu species were the main active sites, and a Zn additive promoted the catalytic reduction activity. In the catalytic hydrogenolysis processes, Cu species were the main catalytic active sites for the cleavage of urethane bonds.

The efficiency of the depolymerization processes for PU1 and PU2 was thoroughly analyzed using gel permeation chromatography (GPC) to measure the materials recovered from the reactions (Fig. 2c). A distinctive observation was that, under CO_2_/H_2_ reaction conditions (Fig. 2c, condition 1), no polymer signals were detected. This highlights the exceptional capability of the designed catalytic system for effective PU degradation. Intriguingly, the process also yielded valuable methylated amines (compounds **b** and **c**, Fig. 2a and Figs S3–S6), which are known to play a crucial role in modulating biological and pharmaceutical activities in life science molecules [40]. The methyl groups in these compounds are believed to originate from CO_2_, as evidenced by experiments under various reaction conditions (Fig. S8). This suggests a sustainable carbon-fixation process, further emphasizing the environmental and industrial relevance of this catalytic method.

To further understand the cleavage of urethane bonds during the hydrogenative depolymerization process, a small model compound (Model 1, propyl *p*-tolylcarbamate) was synthesized (Fig. S9). Since the compound only contains –NH–C(=O)– and –C(=O)–O– bonds that can be cleaved, it can be effectively converted into aromatic amines (4-methylaniline (MA), *N*,4-dimethylaniline (DMA), and 4-methyl-*N*-propylaniline (MPA)) and 1-propanol (Pol) with a total yield of 98% over ZnO-ZrO_2_/Cu in the presence of CO_2_/H_2_ (Fig. 2d and e). The formation of DMA and MPA indicates that N-alkylation occurred during the process. Obviously, the total product yield decreased to 80% under the hydrogen atmosphere (Fig. 2e), suggesting the lower efficiency of catalytic hydrogenation of the ester bonds. In addition, an excessive amount of methanol was used to visualize its effect during catalytic hydrogenation of the urethane bond under an inert atmosphere. In contrast, the total product yield toward MA and Pol was only 47%. The process was achieved along with formation of other *N*-methylated products (DMA and *N,N*,4-trimethylaniline (TMA)) and methanolysis products (methyl *p*-tolylcarbamate (MTC) and *N*-*p*-tolylformamide (TFA)) with a yield of 43% (Fig. 2e). The formations of MTC and TFA can present intermediates for insight into the reaction mechanism during catalytic depolymerization of polyurethane, demonstrating that the effective cleavage of –C(=O)–O– bond precedes the –NH–C(=O)– bond in the presence of methanol. Moreover, the generated *N*-methylated products suggest that excessive methanol in the catalytic system is unfavorable for producing primary amines.

**Figure 3.**
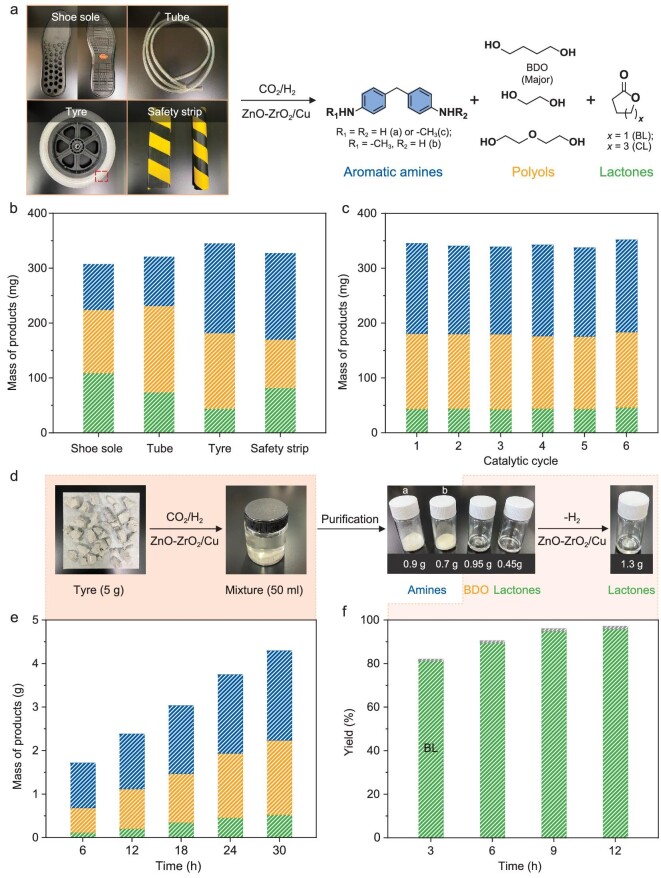
Transformation of real-life PU plastics into valuable chemicals. (a) Schematic reaction pathway for the catalytic degradation of PU plastics. (b) Measured mass of products from hydrogenative degradation of different PU plastics. (c) Repeated catalytic cycles of PU tyre degradation. The catalyst was reactivated after each cycle. (d) Schematic process for transforming 5 g PU tyre into aromatic amines and lactones. (e) Time-dependent mass of products from PU tyre degradation. Conditions in (b, c and e): PU plastics (400 mg or 5 g), ZnO-ZrO_2_/Cu (200 or 500 mg), CO_2_/H_2_ (1/3, v/v, 3 or 4 MPa), THF (30 mL), 200°C, 4 h, 16 h or 30 h. (f) Time-dependent product yield from catalytic dehydrogenation of obtained BDO. Conditions: BDO (0.95 g), ZnO-ZrO_2_/Cu (200 mg), N_2_ (1 MPa), THF (40 mL), 220°C, 12 h. The yields of products in (b, c and e) were calculated from dividing the obtained mass of products by the input amount of PU plastics. The increased mass ratio of all products from CO_2_/H_2_ was <5%. The yields of products in Fig. 2f were calculated from dividing the obtained mol of products by the total input mol of BDO.

Considering that the complex composition and additives in commercial PU plastics could potentially affect the efficiency of our designed catalytic system, we evaluated its robustness using four different commercial PU products: a shoe sole, a tube, a tyre, and a safety strip (Fig. 3a). The catalytic degradation of each of these PU plastics (400 mg each) was assessed, and the results are displayed in Fig. 3b. The total product yields from the catalytic depolymerization were found to be 77%, 80%, 86%, and 82% for the shoe sole, tube, tyre, and safety strip, respectively (Figs S10–S13). These results underscore the effectiveness of our catalytic method in hydrogenatively depolymerizing various commercial PU plastics, despite their differing compositions. Taking the PU tyre as a case study, which contained the highest proportion of urethane linkage (Table S1), we observed consistent catalytic performance over six repeated cycles of transformation (Fig. 3c). This consistent performance is a testament to the excellent stability of our catalytic system, indicating its potential for practical applications in recycling and upcycling commercial PU plastics.

We subsequently conducted a scaled-up deconstruction of a 5 g PU tyre sample to extract basic chemicals for further processing (Fig. 3d). This procedure was executed with exceptional catalytic efficiency, as shown in Fig. 3e. Following purification by flash column chromatography, we isolated 0.90 g of compound **a**, 0.71 g of compound **b**, 0.95 g of BDO, and 0.45 g of lactones (BL and CL) (Fig. 3d, Figs S14 and S15). Given the value of BL as a precursor for biodegradable plastics, we converted the obtained BDO into BL using a straightforward dehydrogenation method [41]. This reaction employed the same ZnO-ZrO_2_/Cu catalyst at 220°C under a nitrogen atmosphere, resulting in a BL yield of 96% over 12 hours (Fig. 3f and Table S2). This BL was then combined with the lactones obtained in the initial step, yielding a total of 1.3 g of lactones (BL/CL ratio of 11/1, mol/mol) (Fig. 3d and Fig. S14). Additionally, both the fresh and spent ZnO-ZrO_2_/Cu catalysts underwent comprehensive characterization using techniques such as XRD, X-ray photoelectron spectroscopy (XPS), X-ray absorption near-edge structure (XANES) spectroscopy, extended X-ray absorption fine-structure (EXAFS), and transmission electron microscopy (TEM) (Figs S1, S16–19, and Table S3). These analyses revealed no significant changes, confirming that the crystalline phase, chemical valence, coordination environment, and morphology of the ZnO-ZrO_2_/Cu catalyst remains highly stable throughout the catalytic processe.

**Figure 4.**
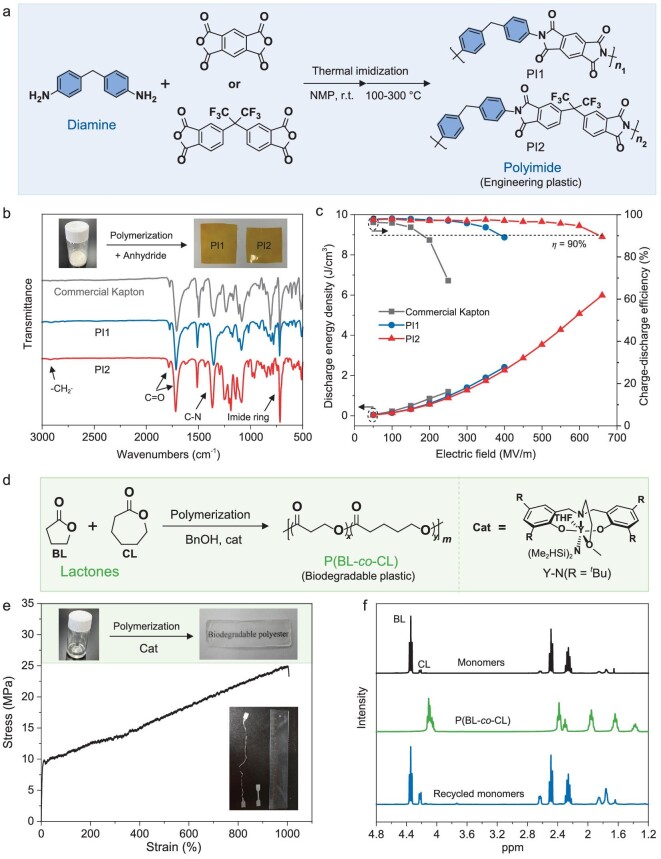
Upcycling of PU waste into valuable and functional polymers. (a) Schematic reaction pathway for the synthesis of polyimide films from obtained aromatic diamine. (b) FT-IR spectra of commercial Kapton, synthesized PI1 and PI2 films. (c) Discharge energy density and charge-discharge efficiency of commercial Kapton, synthesized PI1 and PI2 films at 150°C. (d) Schematic reaction pathway for the synthesis of P(BL-*co*-CL) from obtained lactones. (e) Stress-strain curve (5 mm min^−1^, 5°C) of P(BL-*co*-CL). (f) Overlay of ^1^H-NMR (CDCl_3_, 400 MHz) spectra of starting monomers, synthetic polylactone and recycled monomers.

Aromatic diamines and lactones are key building blocks for diverse applications. In our strategy, the aromatic diamines and lactones obtained from the process were utilized to synthesize two high-value polymers: polyimide (PI) and polylactone (P(BL-*co*-CL)). As depicted in Fig. 1, the 4,4′-methylenedianiline (0.9 g) derived from the PU tyre was reacted with dianhydrides—specifically, pyromellitic dianhydride (PMDA) and 4,4′-(hexafluoroisopropylidiene)diphthalic anhydride (6FDA)—to produce two types of PI films (PI1 and PI2, respectively) as shown in Fig. 4a and b. PI is a crucial engineering plastic, prized for its exceptional thermal stability, electrical insulation, and high mechanical strength [42].

The successful synthesis of PI films was confirmed by infrared (IR) analysis, which revealed characteristic vibration peaks: at 710 cm^−1^ (imide ring), 1365 cm^−1^ (C–N stretch), 1715 cm^−1^ (C=O symmetric stretch), 1780 cm^−1^ (C=O asymmetric stretch), and between 2850 and 2950 cm^−1^ (C–H stretch associated with the –CH_2_– moiety) (Fig. 4b). The properties of the synthesized PI1, PI2, and commercial Kapton films were compared. All films demonstrated excellent solvent resistance, remaining insoluble in various solvents (DCM, NMP, DMF, THF, and DMSO) as shown in Fig. S20. Their thermal stability was assessed using thermal gravimetric analysis (TGA) and dynamic mechanical analysis (DMA) (Fig. S21). The findings showed that the 5% weight loss temperatures and glass transition temperature (*T*_g_) of all films were above 450°C and 300°C, respectively, indicating superior thermal resistance. Additionally, the dielectric properties of the films, including the dielectric constant and loss tangent, were evaluated at a high temperature of 150°C (Fig. S22a). These properties remained stable across a frequency range of 10 to 10^7^ Hz, demonstrating excellent dielectric stability and endurance under high voltage conditions.

The PI1 and PI2 films synthesized in our study show great promise as dielectric materials for high-temperature capacitors. In comparison to commercial Kapton, the synthetic PI1 and PI2 films demonstrated notably higher maximum discharge energy densities (*U*_e_) of 2.4 and 6.0 J cm^−3^, respectively, with charge-discharge efficiencies (*ƞ*) exceeding 90% at 150°C (Fig. 4c). This superior performance is likely attributable to their larger band gap compared to commercial Kapton, as evidenced in Fig. S22b. Furthermore, the PI2 film maintained its excellent performance, with a *U*_e_ of 2.6 J cm^−3^ and *η* >90%, even under more challenging conditions at 200°C (Fig. S23a). This resilience under extreme temperatures highlights the potential of PI2 for advanced applications. Thus, the aromatic diamine recovered from the catalytic degradation of PU plastic waste is not just recycled but significantly upgraded into PI films with competitive properties, as shown in Fig. S23b. This transformation represents a major stride forward in converting waste materials into high-performance products.

In parallel to producing polyimide, we utilized the lactones (1.3 g, BL/CL = 11/1, mol/mol) derived from the PU tyre to synthesize the biodegradable copolyester, P(BL-*co*-CL), through a ring-opening copolymerization reaction (Fig. 4d). Notably, P(BL-*co*-CL) is a green alternative to petrochemical-based polyolefins [43,44], first introduced by Chen *et al.* [45]. We employed an yttrium complex supported with tetradentate aminoalkoxy-bis-phenolate ligands (**Y-N**), a highly efficient catalyst for the ring-opening polymerization of cyclic esters [46], for the copolymerization of the relatively ‘nonstrained’ BL and the more ‘strained’ CL. The copolymerization was conducted at −30°C to minimize the effect of the −*T*Δ*S* term on the Δ*G* of the reaction. After 17 hours, the random copolymer P(BL-*co*-CL) (Mn = 56.3 kg/mol, polydispersity index (Ð) = 1.41) was obtained, with a 73.5% incorporation of BL, alongside a conversion rate of 26.8% for BL and 93.7% for CL (Figs S24–S26). Its thermal properties, analyzed via TGA and differential scanning calorimetry (DSC), revealed a decomposition temperature at 5% weight loss (*T*_d_) of 226°C (Fig. S27a), along with a crystallization temperature (*T*_c_) of −20.9°C and a melting temperature (*T*_m_) of 17.9°C, characterizing it as a semicrystalline random copolymer (Fig. S27b).

The synthetic P(BL-*co*-CL) exhibited excellent mechanical properties, with a toughness of 171 MPa and an impressive elongation at break (*ε*_B_) of over 1000%, as determined by tensile testing of dog-bone-shaped samples at 5°C (Fig. 4e). These values surpass those of the corresponding homopolymers, poly (γ-butyrolactone) (*ε*_B_ <400%) and poly (ε-caprolactone) (*ε*_B_ ≈700%) [47,48]. The copolymer also displayed an ultimate tensile strength (*σ*_B_) of 25.0 MPa and Young's modulus (*E*) of 228 MPa (Fig. 4e), comparable to poly(ethylene terephthalate) and low-density polyethylene [49,50]. Remarkably, the synthetic P(BL-*co*-CL) can be fully recycled back to its original monomers (BL and CL) with a near-quantitative conversion rate of over 98% by heating at 250°C for 12.5 hours in the presence of Y(CH_2_SiMe_3_)_3_(THF)_2_ (5 mol%) (Fig. 3f). The composition of the recycled BL/CL was ∼2.75/1, aligning with the initial composition in P(BL-*co*-CL). Therefore, the lactones sourced from waste PU tyre have been successfully transformed into P(BL-*co*-CL) with outstanding chemical recyclability and ductility.

## CONCLUSION

In conclusion, this study presents an efficient approach for the upcycling of PU waste, utilizing a novel catalytic process that transforms PU into important chemicals and then valuable polymers. By employing a heterogeneous catalytic system combining methanolysis and hydrogenation, we effectively depolymerized PU into aromatic diamines and lactones in CO_2_/H_2_ media. These intermediates were then used to synthesize high-value polymers: polyimide (PI) for advanced engineering applications and polylactone (P(BL-*co*-CL)) as a biodegradable alternative to traditional plastics. The PI films demonstrated exceptional thermal and dielectric properties, while the synthesized P(BL-*co*-CL) exhibited remarkable ductility and recyclability. Our approach not only addresses the valorization of PU plastic waste but also offers a sustainable pathway for converting waste into high-performance materials, contributing to a circular economy.

## Supplementary Material

nwae393_Supplemental_File
