# Supplementary material for: Chemical transformation of polyurethane into valuable polymers
Source: Natl Sci Rev. 2024 Dec 4;12(1):nwae393. doi: 10.1093/nsr/nwae393 (PMC11697979; doi:10.1093/nsr/nwae393)
Supplement: nwae393_Supplemental_File [file nwae393_supplemental_file.pdf]

Supplementary Information for

Chemical Transformation of Polyurethane into Valuable Polymers

Bo Sun[1,#], Jiawei Zou[2,#], Weijie Qiu[1,3,#], Shuheng Tian [1], Maolin Wang[1], Haoyi Tang, Baotieliang Wang[2], Shifang Luan[2], Xiaoyan Tang[1,3], Meng Wang[1,]* and Ding Ma[1,]*

[1]Beijing National Laboratory for Molecular Science, New Cornerstone Science Laboratory, College of Chemistry and Molecular Engineering, Peking University, Beijing 100871, P.R. China

[2]State Key Laboratory of Polymer Physics and Chemistry, Changchun Institute of Applied Chemistry, Chinese Academy of Science, Changchun 130022, P.R. China

[3]Key Laboratory of Polymer Chemistry and Physics of Ministry of Education, Center for Soft Matter Science and Engineering.

[#] equal contribution

Emails of corresponding authors: m.wang@pku.edu.cn; dma@pku.edu.cn

Materials and Methods

1. Chemicals

Catalyst sources: $Cu(NO_3)_2 \cdot 3H_2O$ (99%, Shanghai Aladdin Biochemical Technology Co., Ltd.), $Zr(NO_3)_4 \cdot 5H_2O$ (Energy Chemical Co., Ltd.), $Zn(NO_3)_2 \cdot 6H_2O$ (Sinopharm Chemical), $H_2C_2O_4 \cdot 2H_2O$ (98%, Energy Chemical Co., Ltd.), $Mo_7O_{24} \cdot 4H_2O$ (>99.0%, Sinopharm Chemical).

Polyurethanes and related chemicals: PU1 was synthesized from methylenediphenyl 4,4'-diisocyanate and triethylene glycol. PU2 (particle size: 3 mm, Mw: ca. 296,000, Sigma-Aldrich Co., Ltd.), Model1 was synthesized from *p*-tolyl isocyanate and 1-propanol, 4,4'-diaminodiphenylmethane, 1,4-butanediol, triethylene glycol, diethylene glycol, dipropylene glycol, ethylene glycol, *γ*-butyrolactone, *ε*-caprolactone, *p*-tolyl isocyanate, 1-propanol were all purchased from Shanghai Aladdin Biochemical Technology Co., Ltd. PU plastics, including shoe sole, tube, tyre, and safety strip were all purchased online. Pyromellitic dianhydride (PMDA) and 4,4'-(hexafluoroisopropylidiene)diphthalic anhydride (6FDA) were both purchased from Tokyo Chemical Industry. Initiator benzyl alcohol (BnOH) was purchased from TCI and purified by distillation over CaH2. $Y(CH_2SiMe_3)_3(THF)_2$ and Complex Y-N were prepared according to literature procedures[1,2].

Solvents: tetrahydrofuran (THF), dichloromethane (DCM), *N*-methyl-2-pyrrolidinone (NMP), *N*,*N*-dimethylformamide (DMF), dimethyl sulfoxide (DMSO), THF-d_8, and $CDCl_3$ were bought from Energy Chemical Co., Ltd. Super-dry THF was purchased from J&K.

2. Catalyst Preparation

The $ZnO\text{-}ZrO_2/Cu$ (Zn/Zr/Cu=8/1/2, mol/mol/mol) catalyst was synthesized by co-precipitation method (CP). The oxalic acid was used as the precipitating agent. The detailed information was described as below: 10 mmol $Cu(NO_3)_2 \cdot 3H_2O$, 2.5 mmol $Zr(NO_3)_4 \cdot 5H_2O$ and 1.25 mmol $Zn(NO_3)_2 \cdot 6H_2O$ were dissolved in 100 mL ethanol. Then, the precursor solution was added dropwise into 0.5 M oxalic acid in ethanol solution under vigorous stirring at room temperature. After the co-precipitation process within 2 h, the resultant solid was separated by centrifugation, followed by washing with ethanol and drying at 60 °C for 10 h. The obtained blue powder was calcined in the furnace at 400 °C for 2 h. The obtained black powder was the precursor of $ZnO\text{-}ZrO_2/Cu$. The 200 mg $ZnO\text{-}ZrO_2/Cu$ was heated to 230 °C (2 K min^{-1}) in flowing H_2/Ar (H_2/Ar=1/9, v/v, 60 mL min^{-1}) and held at 230 °C for 2 h to obtain fresh $ZnO\text{-}ZrO_2/Cu$ each time for catalytic hydrogenation of polyurethanes.

3. Evaluation of Catalytic Performance

The catalytic hydrogenations of polyurethanes were carried out in a 100 mL stainless-steel autoclave with mechanical stirring. In a typical procedure, 200 mg activated catalyst was transferred into the autoclave together with 200 mg polyurethane and 30 mL tetrahydrofuran. The autoclave was purged with CO_2/H_2 (CO_2/H_2=1/3, v/v) for five times and the pressure of autoclave was then increased to 3 MPa by filling with CO_2/H_2 (CO_2/H_2=1/3, v/v). The reactor was heated to the desired temperature (about 15 min) and kept at the reaction temperature. After reaction, the liquid phase was separated from the catalyst by centrifugation and the quantitative analysis of liquid products was performed by an Agilent 7820A gas chromatography equipped with a HP-5 capillary column and a flame ionization detector (FID), using tetraethylene glycol dimethyl ether as the internal standard. The quantitative analysis of gas components was performed by a Porapark Q column and a 5A molecular sieve column with a thermal conductivity detector (TCD). Each gas component was calculated using N_2 as the internal standard. The repeated experiments for upcycling of polyurethane tyre were conducted following the procedure above, and the recovered catalyst was reactivated and utilized in next repeated catalytic cycle. The activity of catalyst was measured by testing the yield of degradation products. The product yields and mass balance of catalytic degradation of polyurethanes were calculated using Eqs. (1) and (2), respectively. In most reactions, there were no polyurethanes left, indicated that polyurethanes were completely converted. The product yields of catalytic transformations of Model1 degradation were calculated using Eqs. (2). The product yields of catalytic transformations of BDO dehydrogenation were calculated using Eqs. (3).

$$\text{Yield}i = \frac{\text{Product related to PU monomer}i\ \text{(mg)}}{\text{Polyurethane (input) (mg)}} \times 100\% \qquad (1)$$

$$\text{Mass balance} = \frac{\sum\text{Product related to PU monomer (mg)}}{\text{Polyurethane (input) (mg)}} \times 100\% \qquad (2)$$

$$\text{Yield}i = \frac{\text{Product}i\ \text{(mol)}}{\text{Substrate (input) (mol)*2}} \times 100\% \qquad (3)$$

$$\text{Yield}i = \frac{\text{Product}i\ \text{(mol)}}{\text{Substrate (input) (mol)}} \times 100\% \qquad ()$$

4. Structural Characterization and Synthesis Procedure

X-ray Diffraction (XRD): XRD analysis was carried using PANalytical X'Pert3 Powder X-ray powder diffractometer equipped with a Cu K_α radiation source, at a scan rate of 9 °min^{-1}. The accelerating voltage and current were 40 kV and 40 mA, respectively.

X-ray Potoelectron Spectroscopy (XPS): XPS spectra were measured on an AXIS Supra X-ray photoelectron spectrometer (Kratos Analytical Ltd.) using a monochromatized Al K_α radiation source. The fresh catalyst activated in 10% H_2/Ar was transferred to the chamber of XPS instrument without exposure to air. The spent

catalyst was first dried in a glovebox, and then transferred to the chamber of XPS instrument without exposure to air. The XPS spectra were analyzed using CasaXPS. The binding energy was calibrated by the C 1*s* peak at 284.8 eV.

X-ray Absorption Fine Structure (XAFS) Spectroscopy: XAFS spectra were recorded at the Mo *K*-edge on beamline 1W1B at the Beijing Synchrotron Radiation Facility, which operates at 2.5 GeV with a current of 250 mA. A Si (111) double-crystal monochromator was used. Data were acquired in transmission mode using ionization chambers as detectors. Spectra were acquired under ambient condition.

Scanning Transmission Electron Microscope (STEM): the STEM high-angle annular dark field (STEM-HAADF) images and EDS element maps of the fresh and spend catalysts were measured on a FEI Titian Themis Z microscope at 300 kV. The beam current was approximately 30 pA for HAADF imaging and EDS mapping. The convergence angle used in all STEM characterization was 22.4 mrad and the collection angles of HAADF images was 75-210 mrad. The samples were activated then passivated by 0.5% O_2 in Ar, and dispersed by ethanol before being dropped onto a STEM grid.

Synthesis of Model1: p-tolyl isocyanate (5 g, 37.6 mmol) and 1-propanol (45 g, 750 mmol) were mixed and stirred at 65 °C for 12 h. After completion of the reaction as monitored by GC-MS, the reaction mixture was cooled to room temperature and condensed under reduced pressure, then the crude product was purified by silica gel flash column chromatography using hexane: acetate (3:1) as an eluent to yield 86% (6.2 g) of white solid.

Depolymerization of PU plastics and products separation: the depolymerization procedure was similar to that in the evaluation of catalytic performance. After reaction, collect the reaction solution by centrifuge to remove catalyst. After the reaction solution was condensed under reduced pressure, the crude products were purified by silica gel flash column chromatography using hexane: ethyl acetate (12:1) to ethyl acetate as the gradient eluent to yield 0.9 g aromatic diamine (a), 0.95 g methylated amine (b), 0.95 g BDO, and 0.45 g lactones (BL:CL = 3.2/1)

Synthesis of Polyimide Films: the obtained aromatic diamines (0.9 g, 4.54 mmol) from catalytic degradation of PU waste were first dissolved in NMP (5 ml or 7 ml), then the equimolar amount of PMDA (1.0 g, 4.54 mmol) or 6FDA (2.0 g, 4.54 mmol) was added stepwise into the above solution and stirred at room temperature for 6 h to produce a clear and uniform poly(amic acid) (PAA) solution. Subsequently, the solution was drop cast on clean glass slides, kept in N_2 atmosphere oven at 100 ℃ for 2 h to remove the solvent, and then heated at 150 ℃, 200 ℃, 250 ℃, 300 ℃ for 1 h, respectively. After slowly cooling to room temperature, the observed films could be peeled off from glass substrates in deionized water and then dried in a vacuum oven at 100 ℃ for another 12 h, with a thickness of 10~12 μm.

Characterization of Polyimide Films: the Fourier transform infrared (FT-IR) spectra were measured on a Bruker alpha. Thermal gravity analysis (TGA, TA TGA Q50) and dynamic mechanical analysis (DMA, METTLER TOLEDO DMA/SDTA861e) were performed in nitrogen atmosphere, during which the heating rate was set as

10 ℃ min^{-1} and 5 ℃ min^{-1}, respectively. PerkinElmer Lambda 35 instrument was used for the measurement of UV-vis spectra and the bandgap of PI films was obtained according to the relationship between $(ahv)^2$ and *hv* by Tauc plot. Dielectric constant and dielectric loss were measured using an Agilent LCR meter (4294A), and Cu electrodes (diameter 3.4 mm and thickness 50 nm) were deposited on both sides of the PI films for the electrical measurements. The energy storage performance of PI films was collected using a modified Sawyer-Tower circuit with a high-voltage amplifier system at 150 ^oC or 200 ^oC.

Synthesis of Polylactone: polymerizations were performed in 5 mL glass reactors inside the inert glovebox at -30 ℃. The solution of 6.8 μmol catalyst was premixed with the initiator 6.8 μmol BnOH, stirred for 10 min and then the mixture was added to the vigorously stirred precooled monomer solution with 1.3 g monomers (BL/CL/Y-N/Initiator = 2000/200/1/1, BL = 10 M in THF, V_{THF} = 170 μL). After the predetermined time, the polymerization was quenched by addition of benzoic acid/chloroform (10 mg mL^{-1}) and a 0.02 mL of aliquot was taken from the reaction mixture and prepared for ^1H NMR analysis to obtain the percent monomer conversion data. The quenched mixture was then precipitated into cold methanol while stirring, centrifuged and removed clear liquid. After dissolving participate with DCM, this procedure was repeated twice to ensure any catalyst residue or unreacted monomer was removed. The polymer was dried in a vacuum oven at R.T. to a constant weight.

Characterization of Polylactone: ^1H-NMR (400 MHz) spectra was recorded on Bruker AVANCE III 400 spectrometer. Chemical shifts for ^1H and ^{13}C spectra were referenced to internal solvent resonances and are reported as parts per million compared to $SiMe_4$. Measurements of polymer weight-average molar mass (M_w), number-average molar mass (M_n), and dispersity ($Đ = M_w/M_n$) were performed via size exclusion chromatography (SEC). The SEC measurements were performed on an Agilent 1260 Infinity II instrument equipped with two Agilent PL gel columns (MIXED-C, MIXED-D) and a RI detector. HPLC grade THF was used as the eluent with a flow rate of 1.0 mL min^{-1} at 35 ℃. M_n and $Đ$ were obtained on Agilent software under a calibration curve from polystyrene standards. TGA was performed on a TA Instrument Q600 analyzer. Samples were heated from ambient temperatures to 600 ℃ at a heating rate of 10 ℃ min^{-1}. $T_{d,5\%}$ values were obtained from wt% vs. temperature (℃) plots and defined by the temperature of 5% weight loss. Differential scanning calorimetry (DSC) analyses were performed on a TA Instrument DSC 250, T_m and T_g values were obtained from a second scan after the thermal history was removed from the first scan. The first heating rate was 20 ℃ min^{-1}, while the cooling rate was 5 ℃ min^{-1} and the second heating rate was 10 ℃ min^{-1}. Tensile stress/strain testing was performed by 3365 (50 N load cell) universal testing system on dog-bone-shaped test specimen generated via slow-solvent evaporation. Test specimens were stretched at a strain rate of 5 mm min^{-1} at approximately 5 ℃.

Catalytic Depolymerization of Synthetic Polylactone: A 10 mL Schlenk tube containing the purified polymer P(BL-*co*-CL) (20 mg) and 5 mol% catalyst was sealed and heated to 250 °C under nitrogen atmosphere. After the predetermined time, the mixture was slowly cooled and quenched by addition of benzoic acid/chloroform

(10 mg mL^{-1}). The overall mixture including the colorless liquid appeared at the top of the tube wall and the solid remaining at the tube bottom are analyzed by ^{1}H-NMR together, confirming that two monomers were cleanly recycled in near-quantitative conversion (> 98%).

Supplementary Figures and Tables

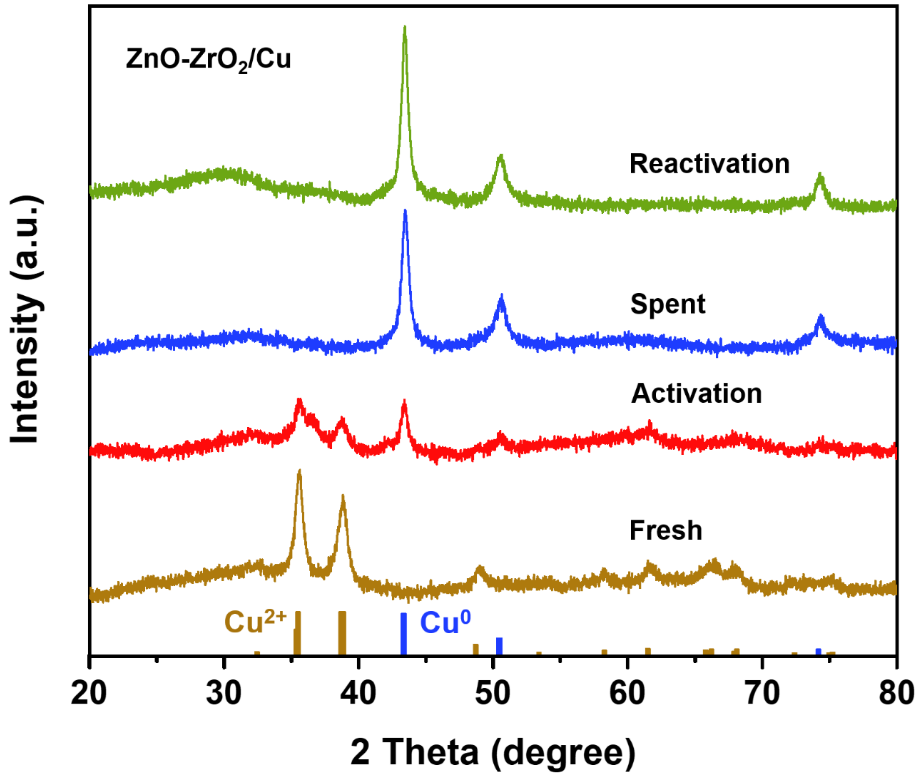

Figure S1. Powder XRD spectra of ZnO-ZrO_2/Cu catalyst at different stage.

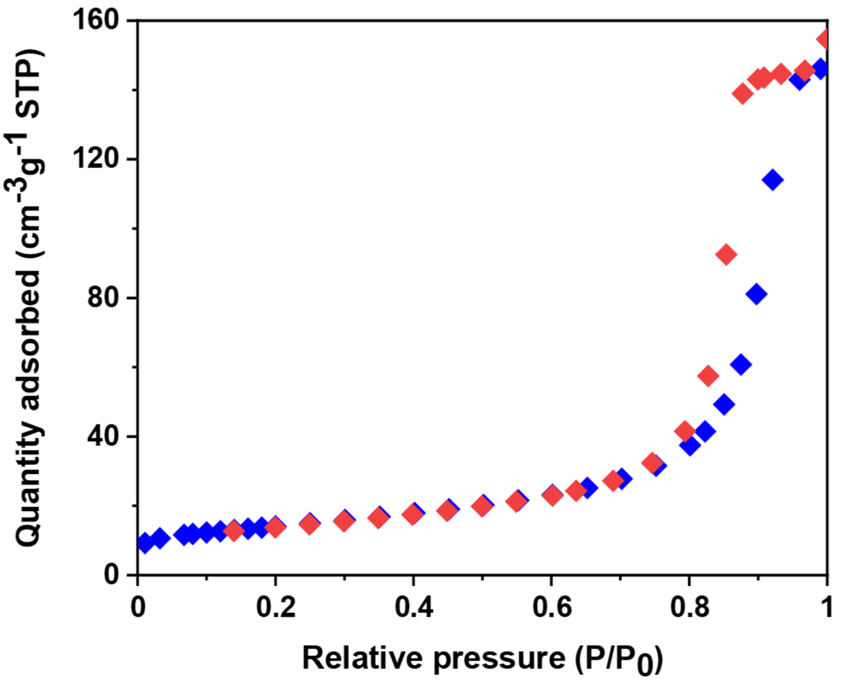

Figure S2. Nitrogen adsorption-desorption isotherms of ZnO-ZrO_2/Cu. The surface BET area is 49.6 ± 0.2 m^2 g^{-1}.

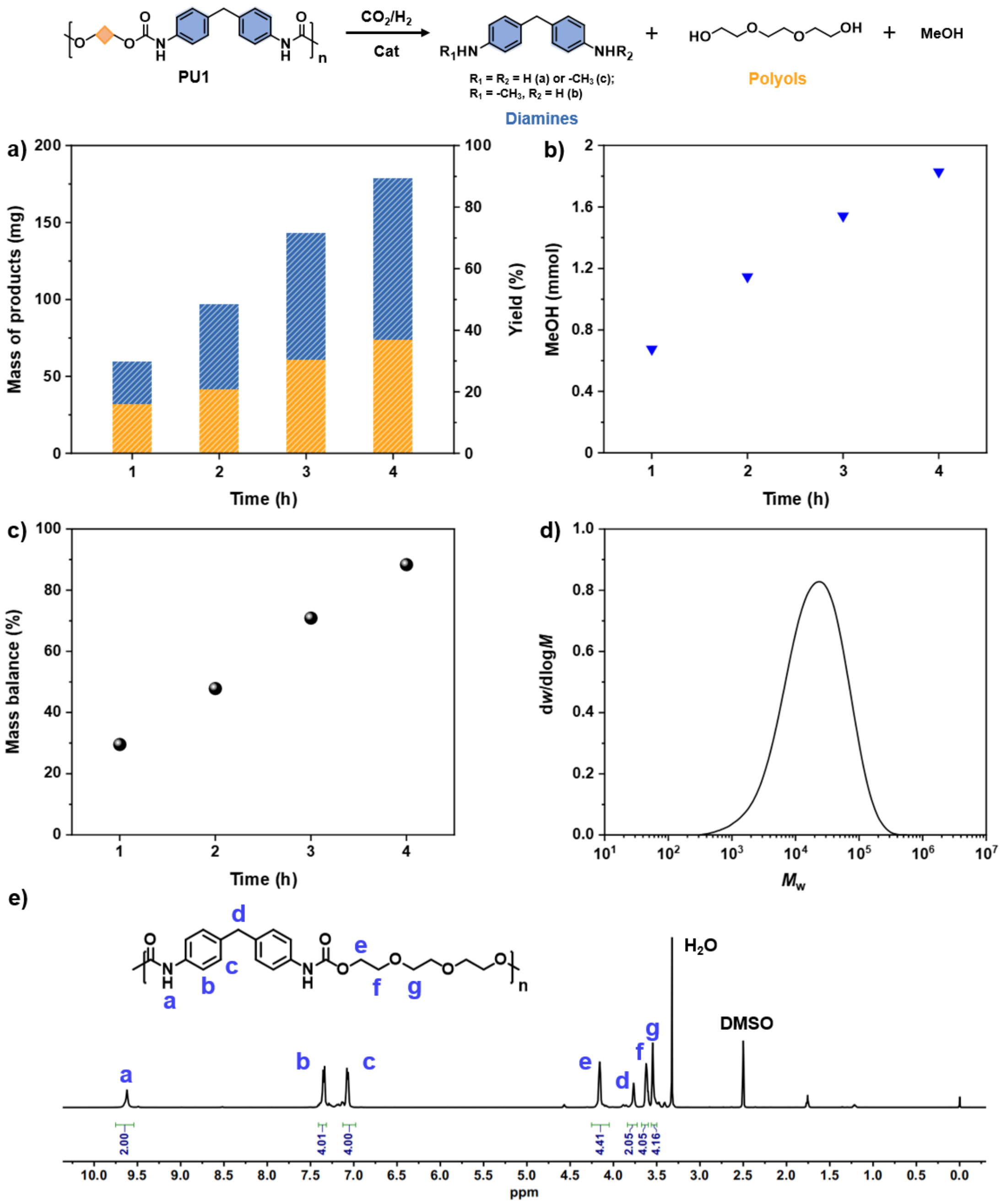

Figure S3. Time-dependent yield of products from catalytic hydrogenation of PU1. Liquid product analysis (a) and (b), and mass balance (c) from catalytic hydrogenation of PU1 over ZnO-ZrO_2/Cu. Reaction conditions: 200 mg PU1, 200 mg ZnO-ZrO_2/Cu, and 30 mL THF were stirred in an autoclave with 3 MPa (CO_2/H_2 = 1/3, v/v) at 200 ℃. The ratio of Products: a/b/c = 3.2/1.3/1, mol/mol. (d) Molecular weight distribution of PU1 measured by Gel Permeation Chromatography (GPC) (Mw: ca. 32,711). (e) ^{1}H-NMR of PU1 in DMSO-d_6.

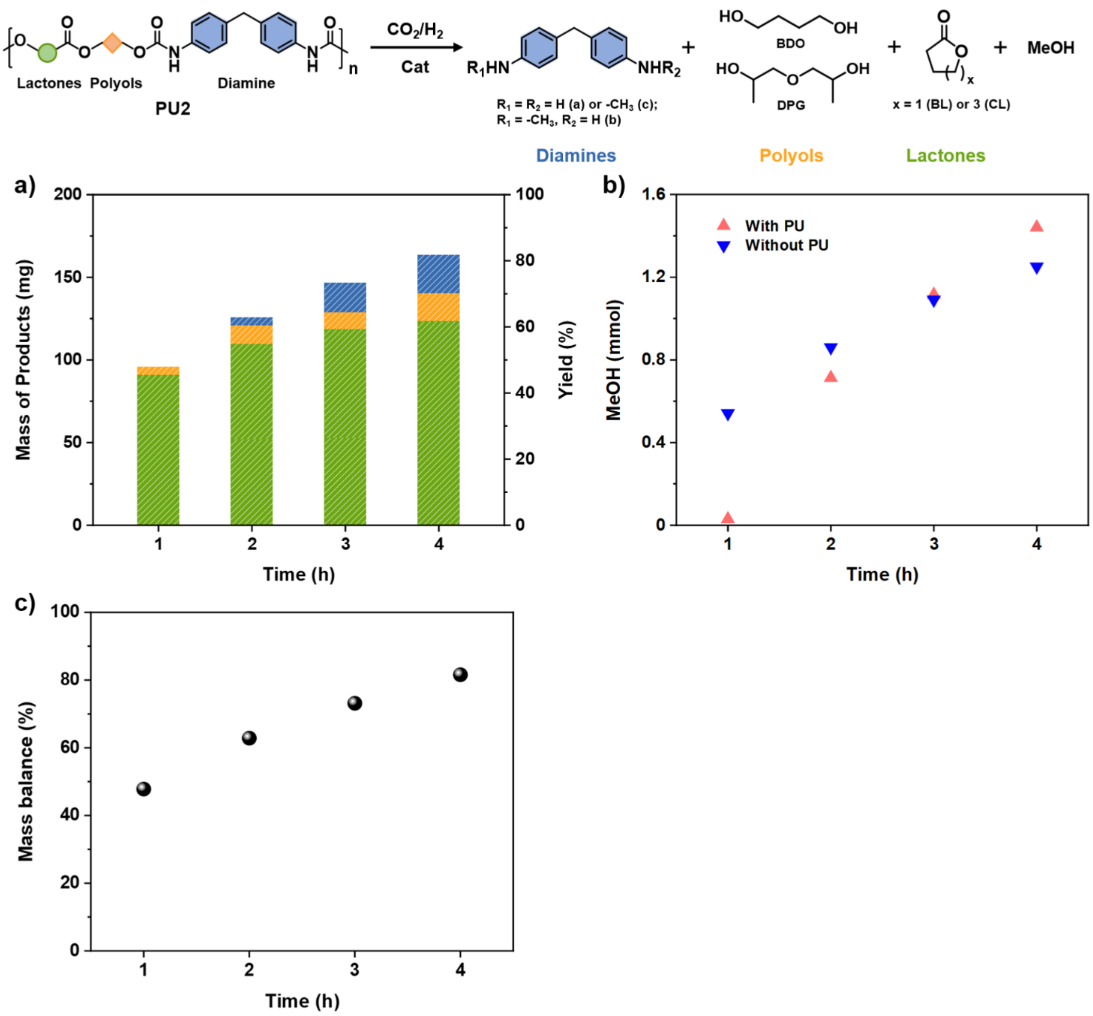

Figure S4. Time-dependent yield of products from catalytic hydrogenation of PU2. Liquid product analysis (a) and (b), and mass balance (c) from catalytic hydrogenation of PU2 over ZnO-ZrO_2/Cu. Reaction conditions: 200 mg PU2 or none, 200 mg ZnO-ZrO_2/Cu, and 30 mL THF were stirred in an autoclave with 3 MPa (CO_2/H_2 = 1/3, v/v) at 200 °C. The ratio of Products: a/b/c = 4/6/1; BDO/BPG = 1.6/1; BL/CL = 1.3/1, mol/mol.

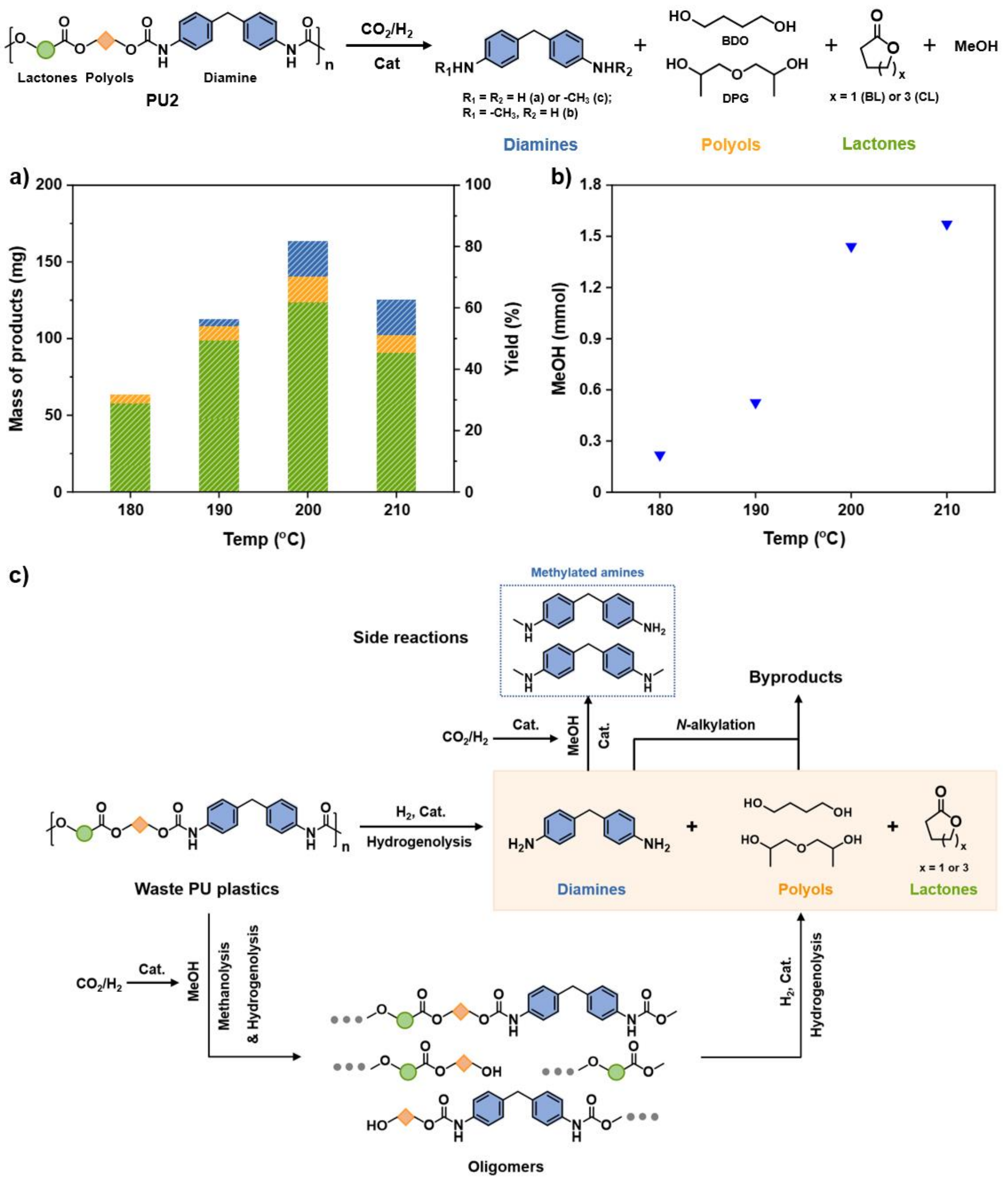

Figure S5. Catalytic hydrogenation of PU2 under different temperature conditions. Liquid product analysis (a) and (b) from catalytic hydrogenation of PU2 over ZnO-ZrO_2/Cu. Reaction conditions: 200 mg PU2, 200 mg ZnO-ZrO_2/Cu, and 30 mL THF were stirred in an autoclave with 3 MPa (CO_2/H_2 = 1/3, v/v) for 4 h. (c) The detailed reaction pathway for the depolymerization process of PU2.

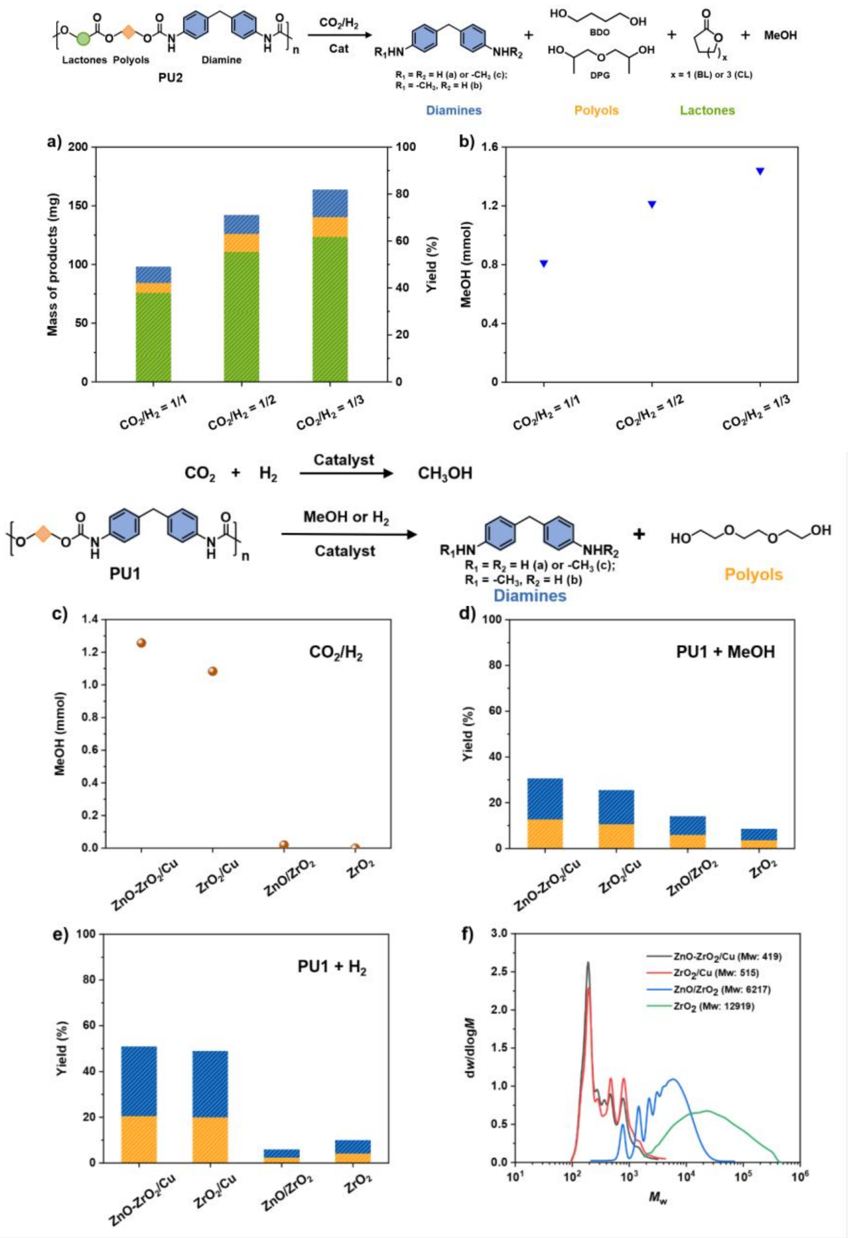

Figure S6. Catalytic hydrogenation of PU2 under different atmosphere conditions and the identification of catalytic active sites for different processes. Liquid product analysis (a) and (b) from catalytic hydrogenation of PU2 over ZnO-ZrO_2/Cu. Reaction conditions: 200 mg PU2, 200 mg ZnO-ZrO_2/Cu, and 30 mL THF were stirred in an autoclave with 3 MPa gas at 200 ℃ for 4 h. (c) The reduction of CO_2 to methanol over different catalysts. Conditions: catalyst (200 mg), CO_2/H_2 (1/3, v/v, 3 MPa), THF (30 mL), 200 ℃, 4 h. (d) Yield of products from catalytic methanolysis of PU1. Conditions: PU1 (200 mg), catalyst (200 mg), MeOH (250 μL), THF (30 mL), 200 ℃, 4 h. (e) Yield of products from catalytic hydrogenolysis of PU1. Conditions: PU1 (200 mg), catalyst (200 mg), H_2 (2.3 MPa), THF (30 mL), 200 ℃, 4 h. (f) GPC measurements for the different reaction conditions in (d).

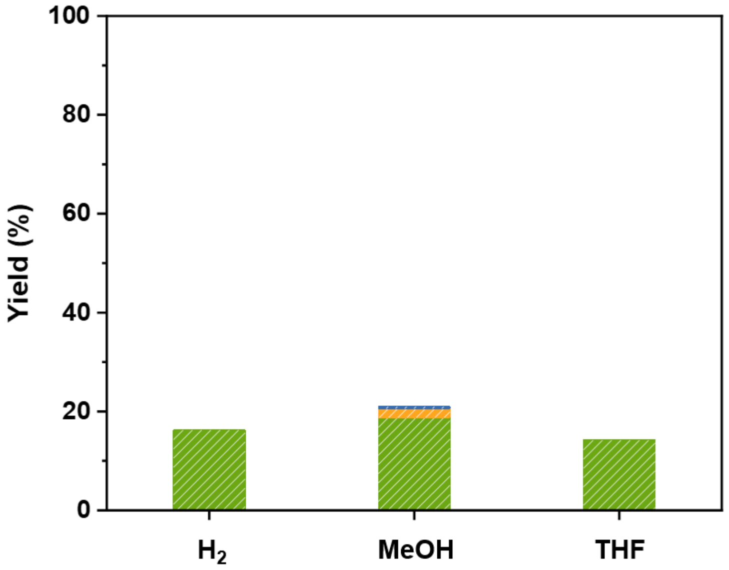

Figure S7. Degradation of PU2 without catalyst under different conditions. Liquid product analysis from degradation of PU2 without catalyst. Reaction conditions: 200 mg PU2, and 30 mL THF were stirred in an autoclave (with 2.2 MPa H_2; 1.5 mmol MeOH; 1 MPa N_2) at 200 ℃ for 4 h.

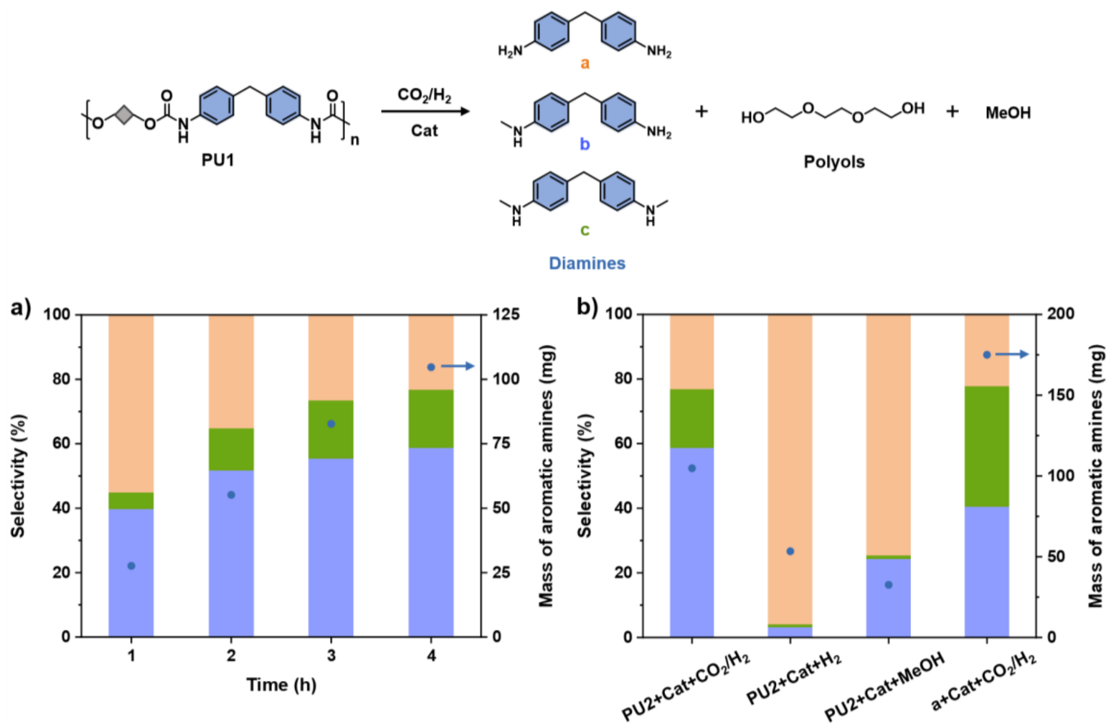

Figure S8. The products selectivity towards aromatic amines from catalytic hydrogenation of PU1 under different conditions. The selectivity analysis of aromatic amines with time changing (a) and under different reaction conditions (b) over ZnO-ZrO_2/Cu. Reaction conditions: 200 mg PU1 or 200 mg **a**, 200 mg ZnO-ZrO_2/Cu, and 30 mL THF were stirred in an autoclave at 200 °C with (a) 3 MPa (CO_2/H_2 = 1/3, v/v) or (b) 3 MPa (CO_2/H_2 = 1/3, v/v); 2.2 MPa H_2; 1MPa N_2, 2 mmol MeOH; 3 MPa (CO_2/H_2 = 1/3, v/v).

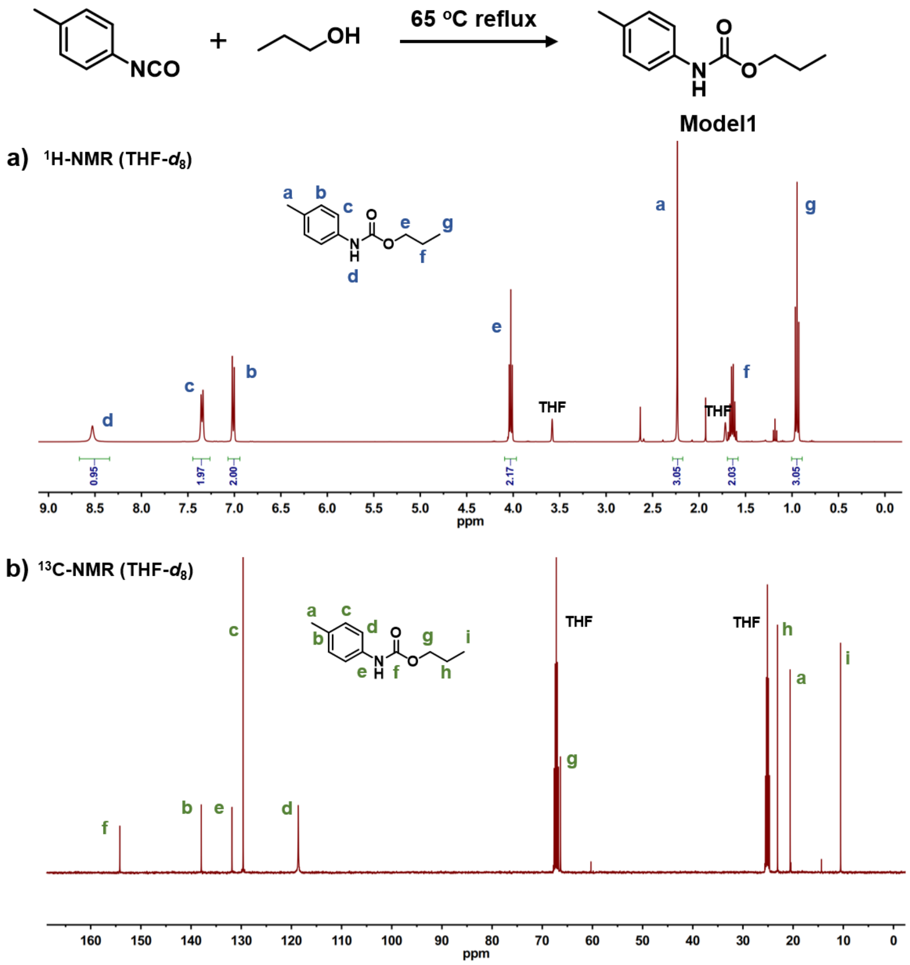

Figure S9. ^{1}H-NMR (a) and ^{13}C-NMR (b) of synthesized **Moldel1** after purification by flash gel chromatography.

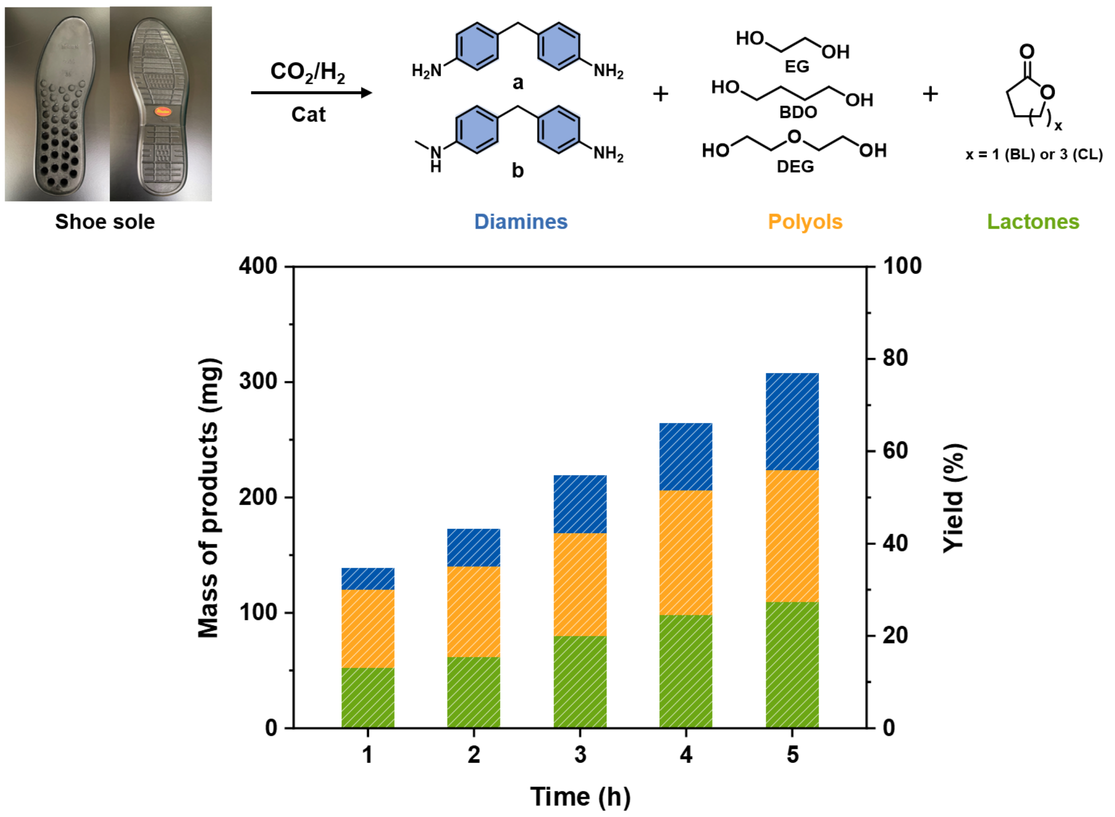

Figure S10. Time-dependent yield of products from catalytic hydrogenation of PU shoe sole. Liquid product analysis from catalytic hydrogenation of PU shoe sole over ZnO-ZrO_2/Cu. Reaction conditions: 400 mg PU shoe sole, 200 mg ZnO-ZrO_2/Cu, and 30 mL THF were stirred in an autoclave with 3 MPa (CO_2/H_2 = 1/3, v/v) at 200 °C. The ratio of Products: a/b = 1.5/1, EG/BDO/DEG = 4.4/8.8/1, BL/CL = 26/1, mol/mol.

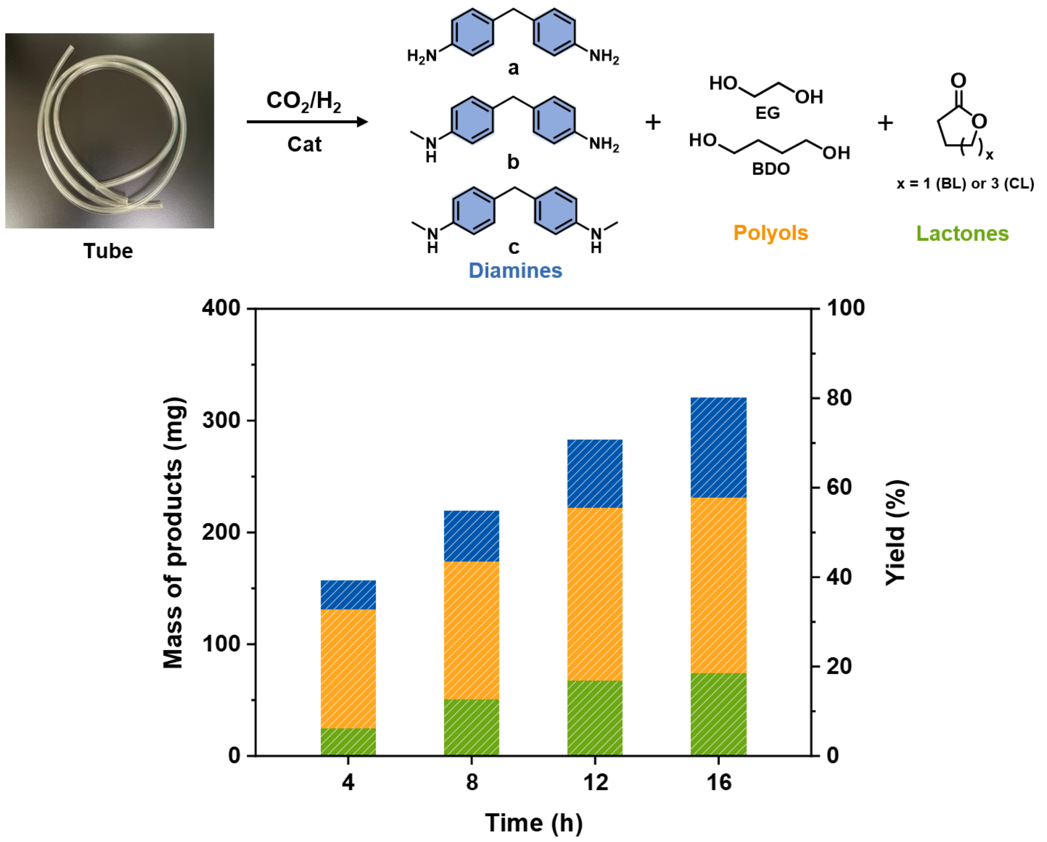

Figure S11. Time-dependent yield of products from catalytic hydrogenation of PU tube. Liquid product analysis from catalytic hydrogenation of PU tube over ZnO-ZrO_2/Cu. Reaction conditions: 400 mg PU tube, 200 mg ZnO-ZrO_2/Cu, and 30 mL THF were stirred in an autoclave with 3 MPa (CO_2/H_2 = 1/3, v/v) at 200 °C. The ratio of Products: a/b/c = 6/7/1, EG/BDO = 1/3, BL/CL = 2/1, mol/mol.

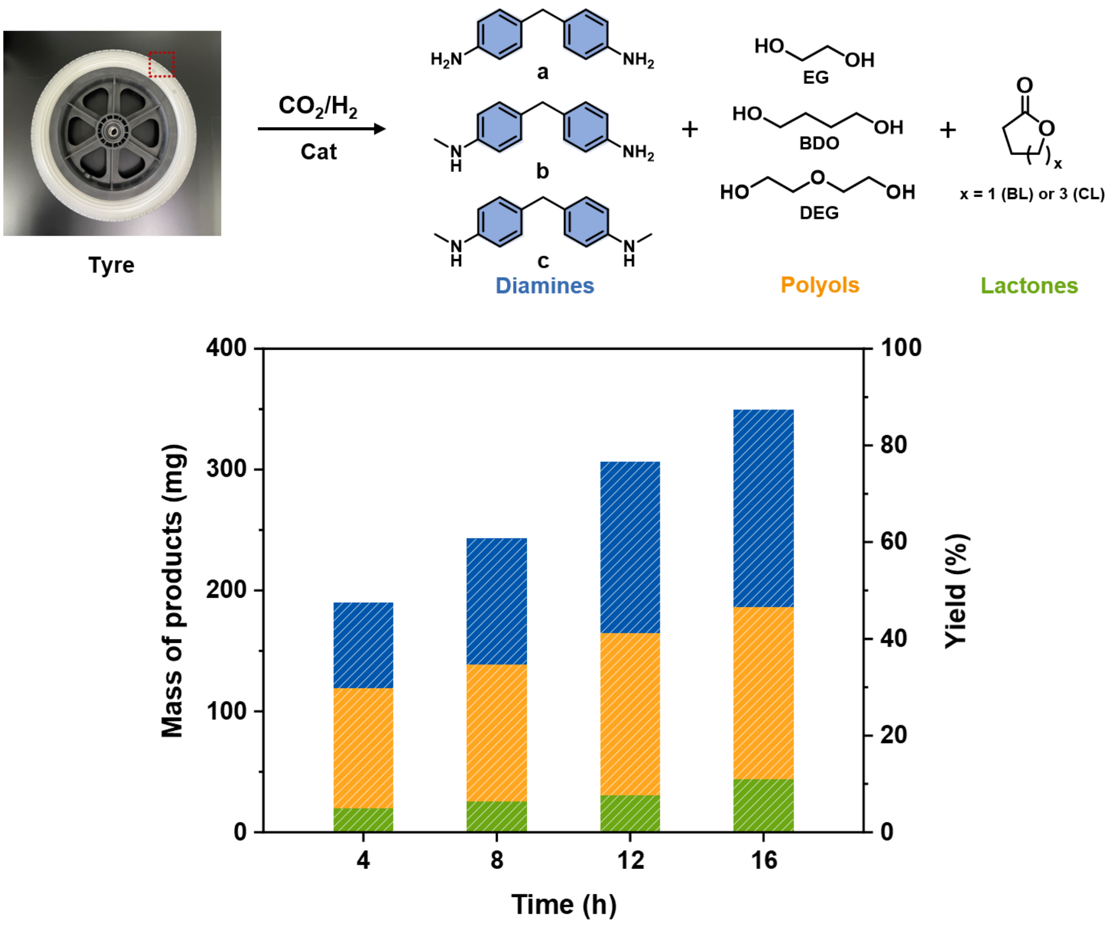

Figure S12. Time-dependent yield of products from catalytic hydrogenation of PU tyre. Liquid product analysis from catalytic hydrogenation of PU tyre over ZnO-ZrO_2/Cu. Reaction conditions: 400 mg PU tyre, 200 mg ZnO-ZrO_2/Cu, and 30 mL THF were stirred in an autoclave with 3 MPa (CO_2/H_2 = 1/3, v/v) at 200 °C. The ratio of Products: a/b/c = 5/5/1, EG/BDO/DEG = 1.7/4.6/1, BL/CL = 3/1, mol/mol.

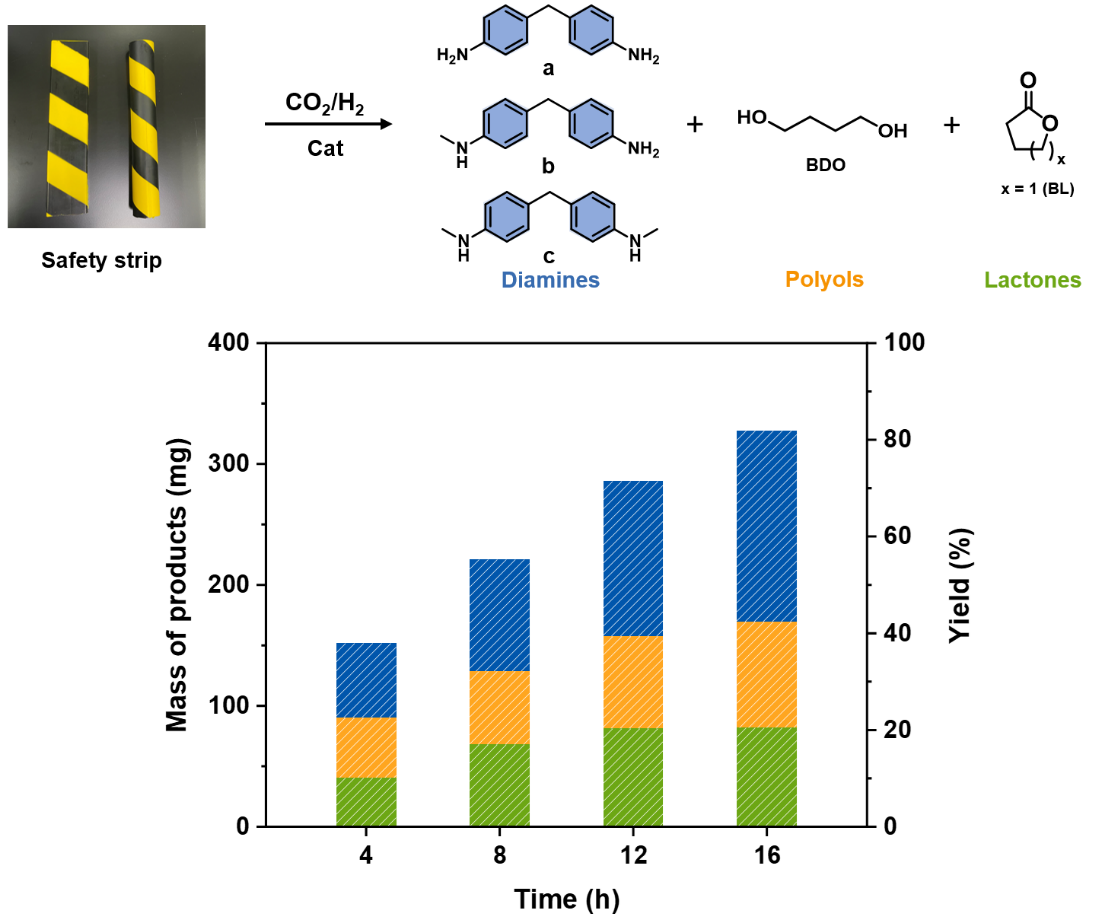

Figure S13. Time-dependent yield of products from catalytic hydrogenation of PU safety strip. Liquid product analysis from catalytic hydrogenation of PU safety strip over ZnO-ZrO_2/Cu. Reaction conditions: 400 mg PU safety strip, 200 mg ZnO-ZrO_2/Cu, and 30 mL THF were stirred in an autoclave with 3 MPa (CO_2/H_2 = 1/3, v/v) at 200 °C. The ratio of Products: a/b/c = 1/16/5, mol/mol.

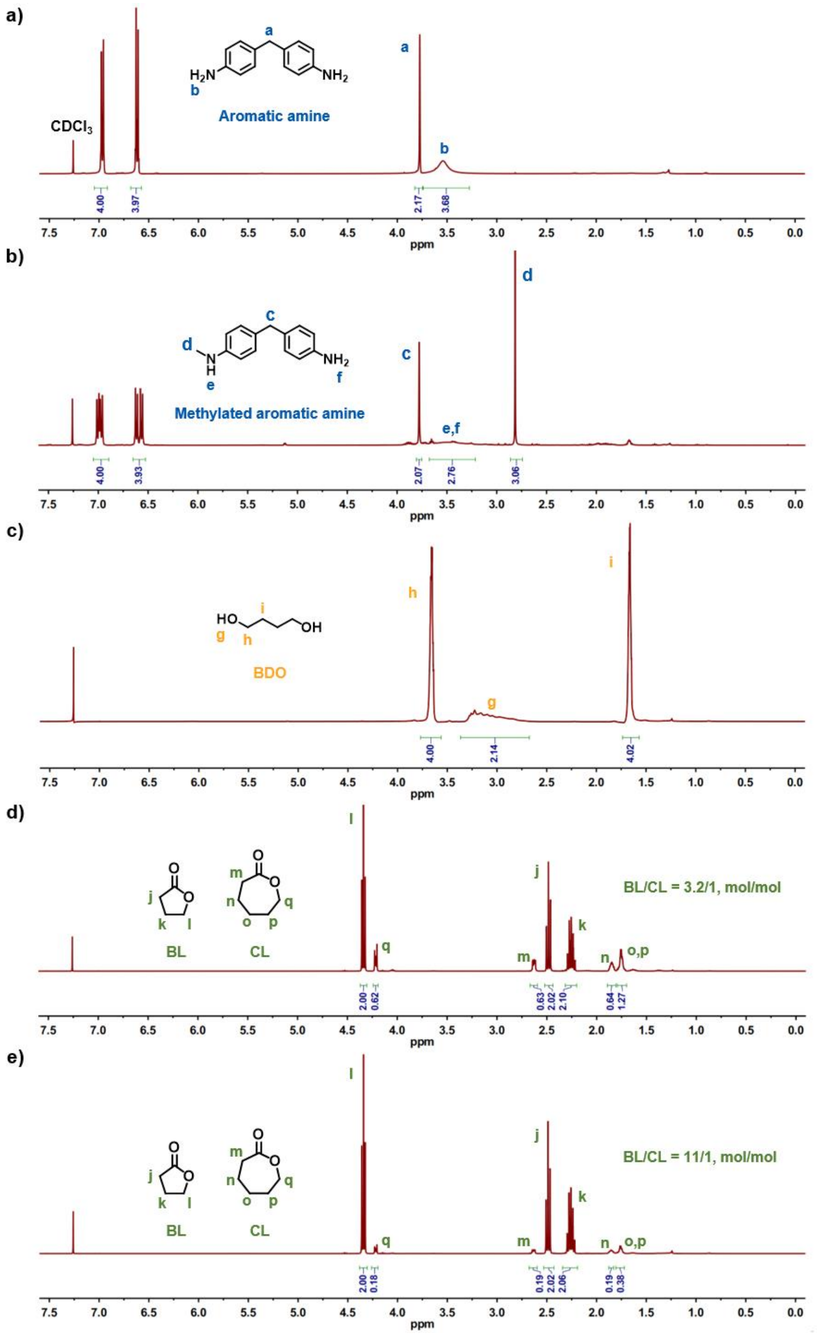

Figure S14. ^{1}H-NMR ($CDCl_3$) of obtained (a) aromatic amine, (b) methylated aromatic amine, (c) 1,4-butanediol (BDO), (d) lactones (BL/CL = 3.2/1, mol/mol) from catalytic upcycling of polyurethane and subsequent separation process via flash gel chromatography, and (e) combined lactones (BL/CL = 11/1, mol/mol), including lactones in (d) and converted γ-butyrolactone (BL) form catalytic dehydrogenation of BDO with subsequent separation process via flash gel chromatography.

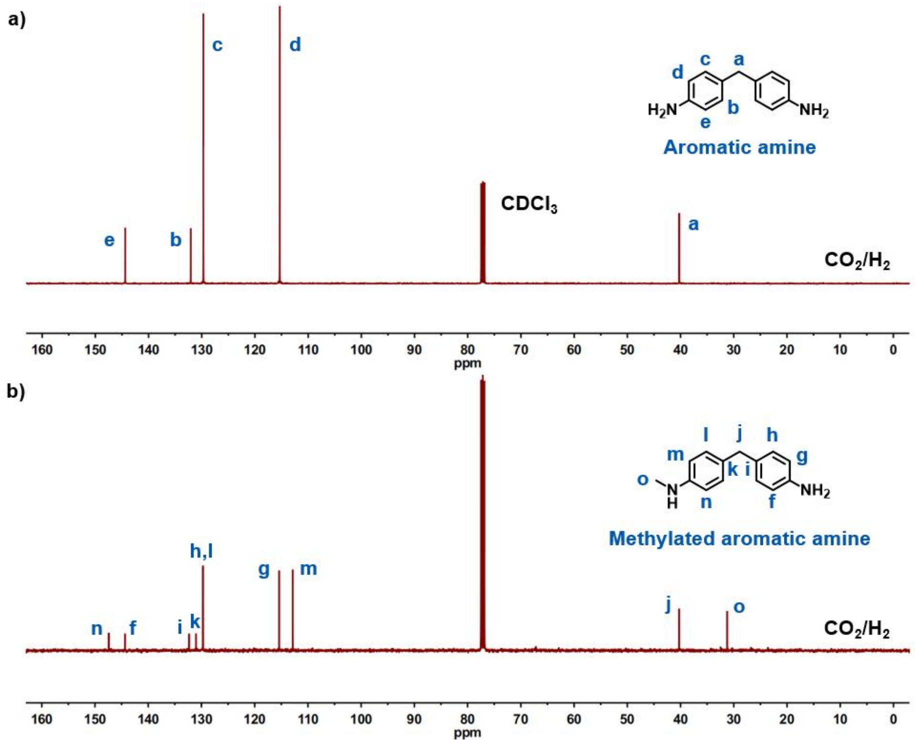

Figure S15. ^{13}C-NMR ($CDCl_3$) of obtained (a) aromatic amine, and (b) methylated aromatic amine from catalytic upcycling of polyurethane in presence of CO_2/H_2 and subsequent separation process via flash gel chromatography.

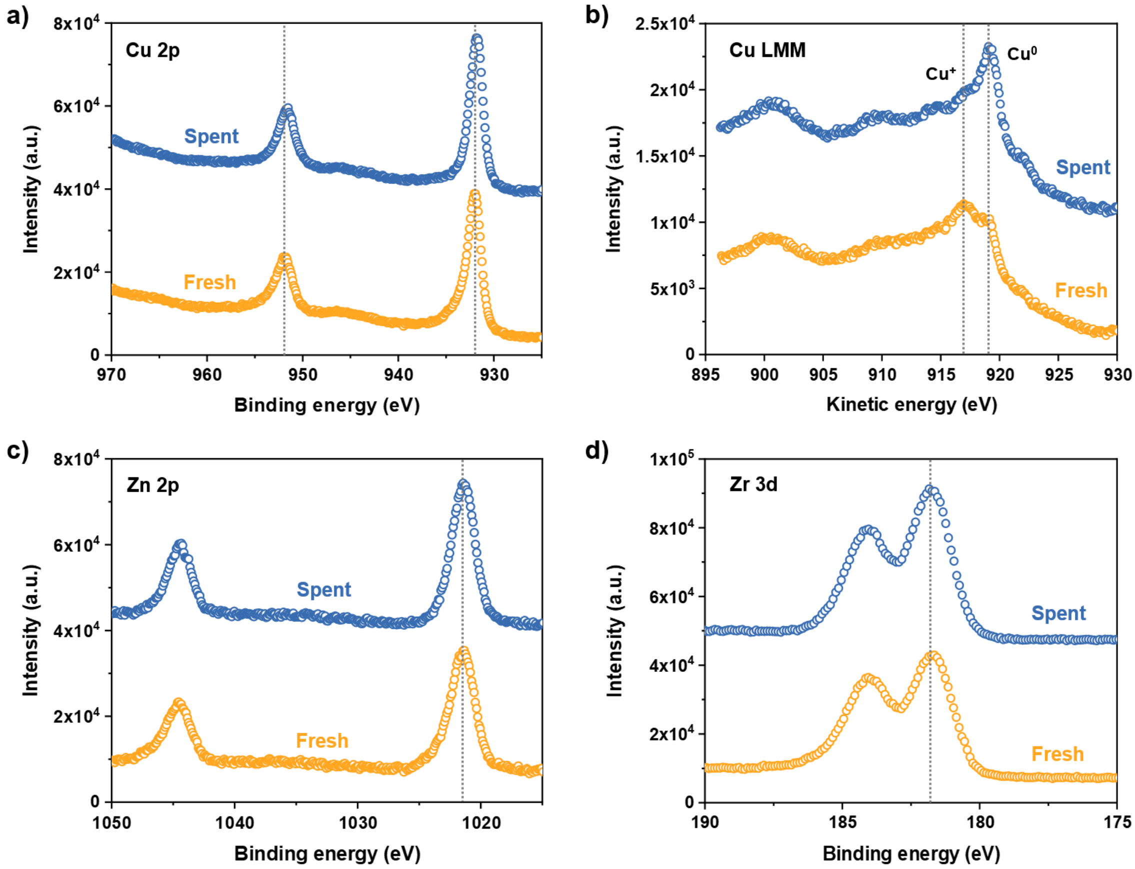

Figure S16. XPS spectra of (a) Cu *2p*, (b) Cu LMM auger, (c) Zn *2p*, and (d) Zr *3d* regions from fresh and spent ZnO-ZrO_2/Cu.

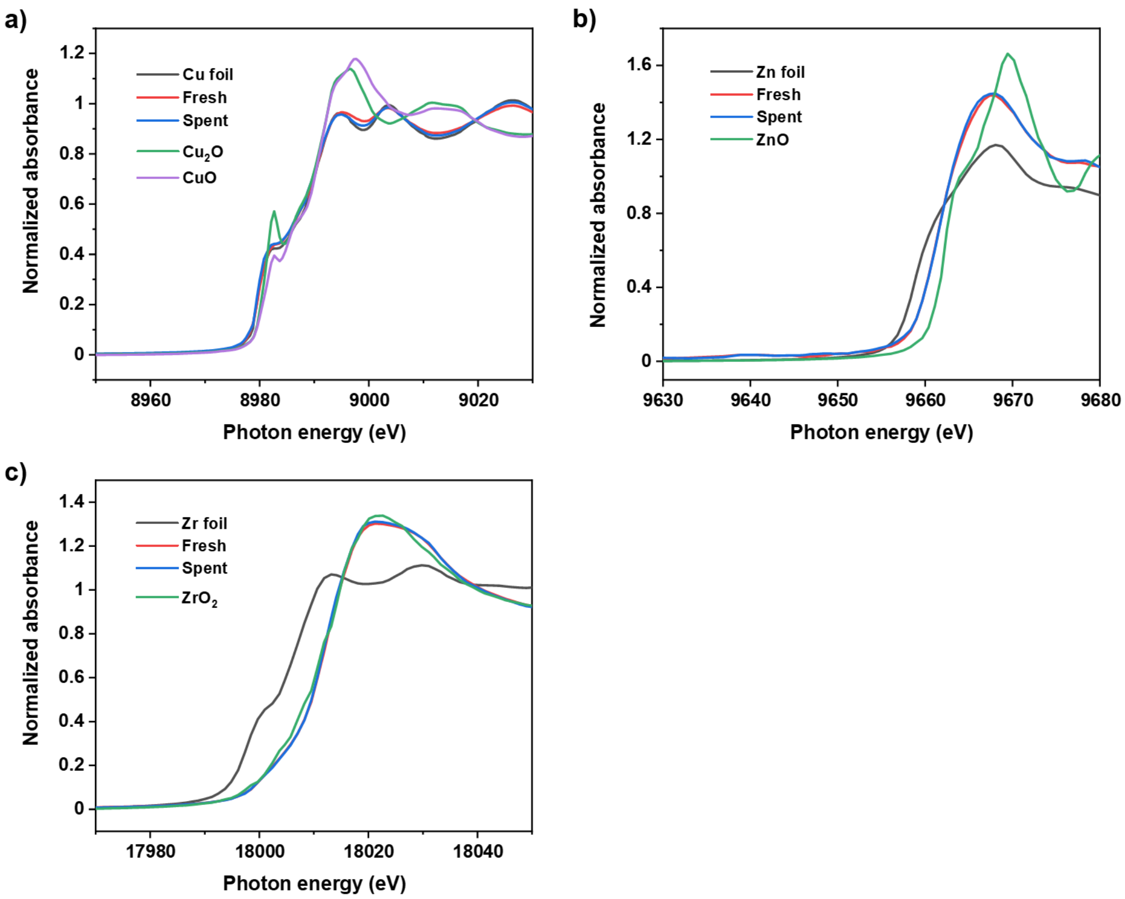

Figure S17. (a) Cu *K*-edge XANES for fresh, and spent ZnO-ZrO_2/Cu and three model compounds: Cu foil, Cu_2O, CuO. (c) Zn *K*-edge XANES for fresh, and spent ZnO-ZrO_2/Cu and two model compounds: Zn foil, ZnO. (b) Zr *K*-edge XANES for fresh, and spent ZnO-ZrO_2/Cu and two model compounds: Zr foil, ZrO_2.

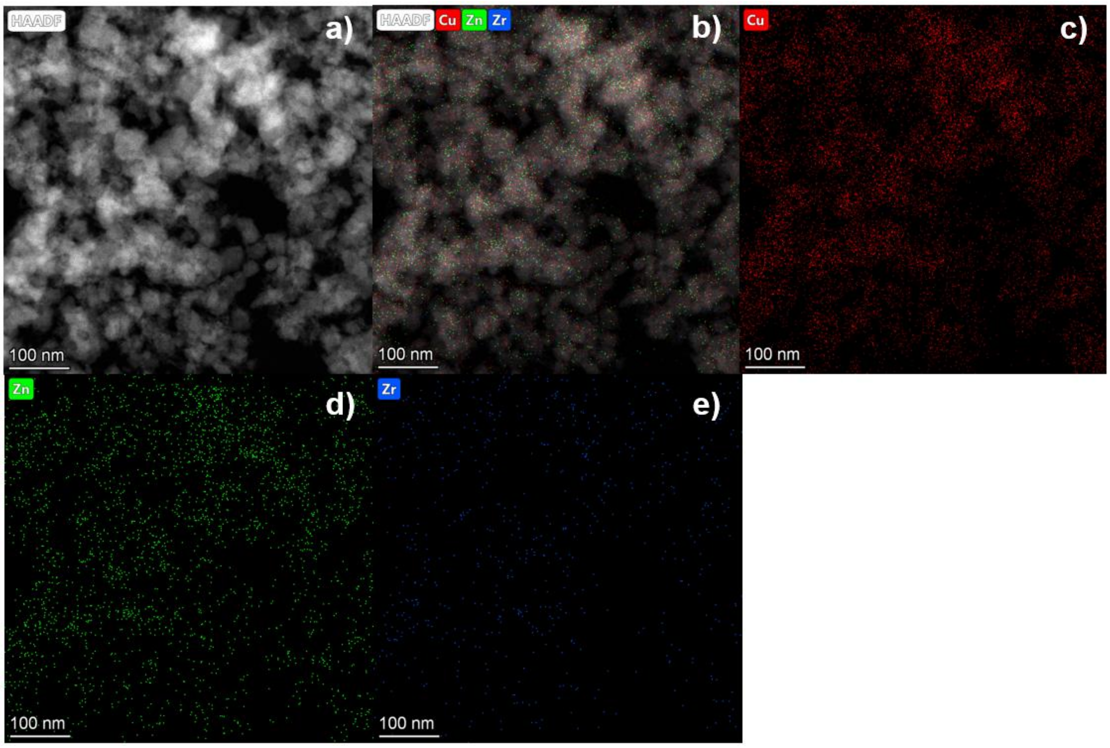

Figure S18. Structural characterization of fresh ZnO-ZrO_2/Cu. (a) HAADF-STEM image of fresh ZnO-ZrO_2/Cu. (b) Overlay of Cu, Zn and Zr EDS maps with the simultaneously recorded HAADF-STEM image. EDS maps of (c) Cu, (d) Zn, and (e) Zr.

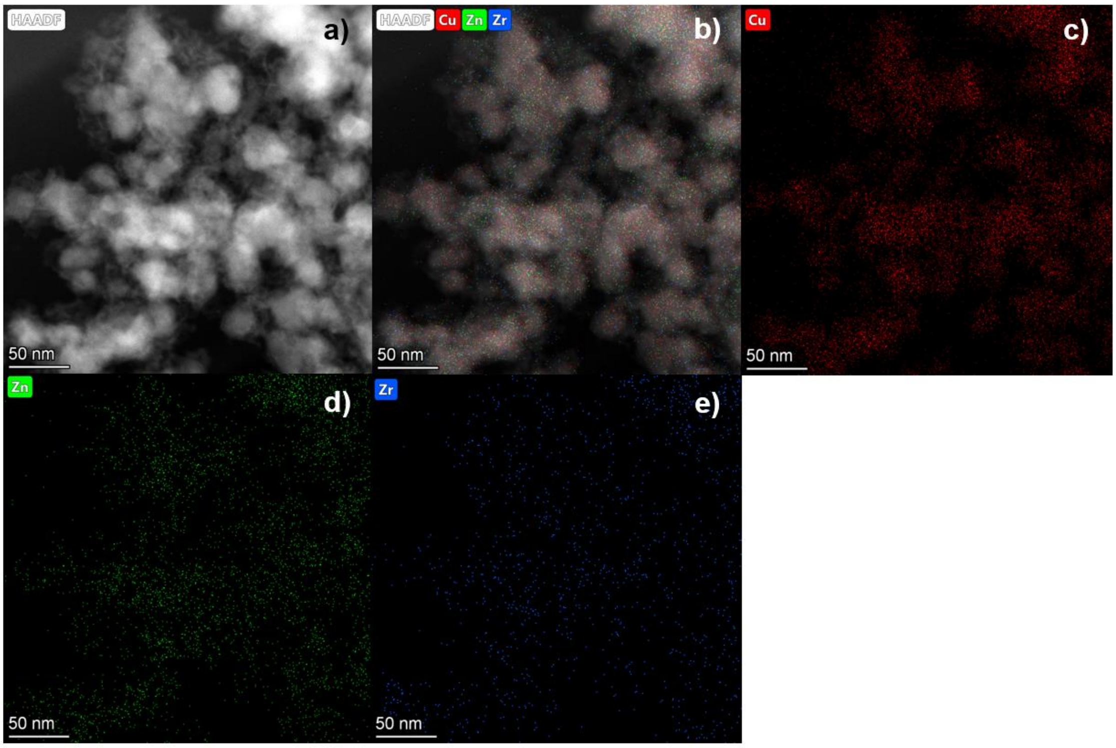

Figure S19. Structural characterization of spent ZnO-ZrO_2/Cu. (a) HAADF-STEM image of spent ZnO-ZrO_2/Cu. (b) Overlay of Cu, Zn and Zr EDS maps with the simultaneously recorded HAADF-STEM image. EDS maps of (c) Cu, (d) Zn, and (e) Zr.

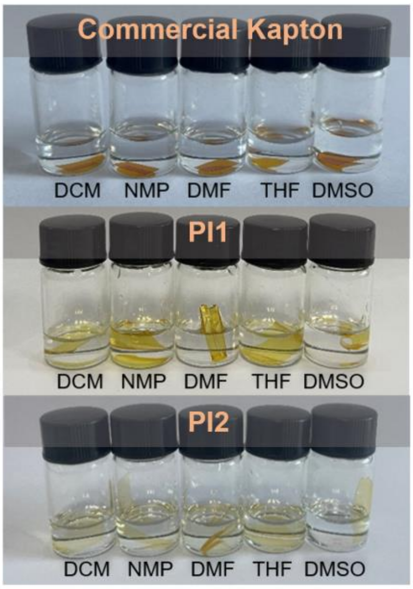

Figure S20. Solubility tests of commercial Kapton, synthetic PI1 and PI2 films in different solvents (including DCM, NMP, DMF, THF, and DMSO), the results indicated that the synthesized PI1 and PI two films exhibited excellent solvent resistance property.

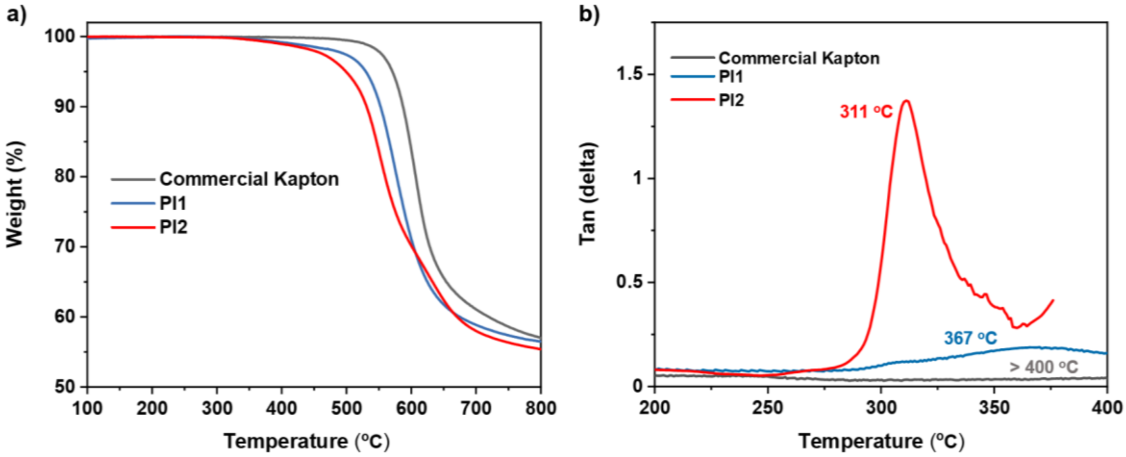

Figure S21. (a) Measurements of thermogravimetric analysis (TGA) for commercial Kapton, synthesized PI1 and PI2 films with a heating rate of 10 ℃ under N_2 atmosphere. (b) Loss factor curves from commercial Kapton, synthesized PI1 and PI2 films.

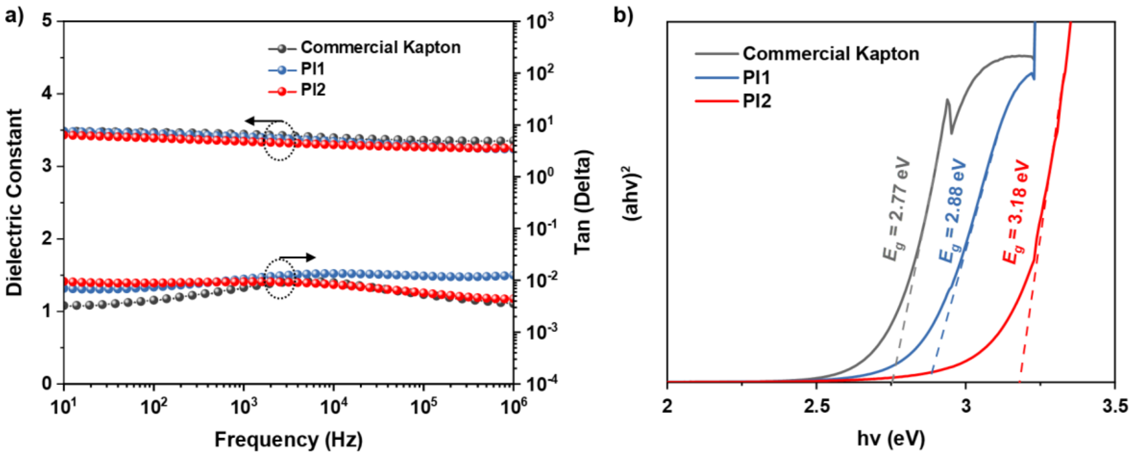

Figure S22. (a) Measurements of frequency-dependent dielectric constant and tan (delta) of commercial Kapton, synthesized PI1 and PI2 films at 150 °C. (b) bandgaps (E_g) based on UV-vis results of commercial Kapton, synthesized PI1 and PI2 films.

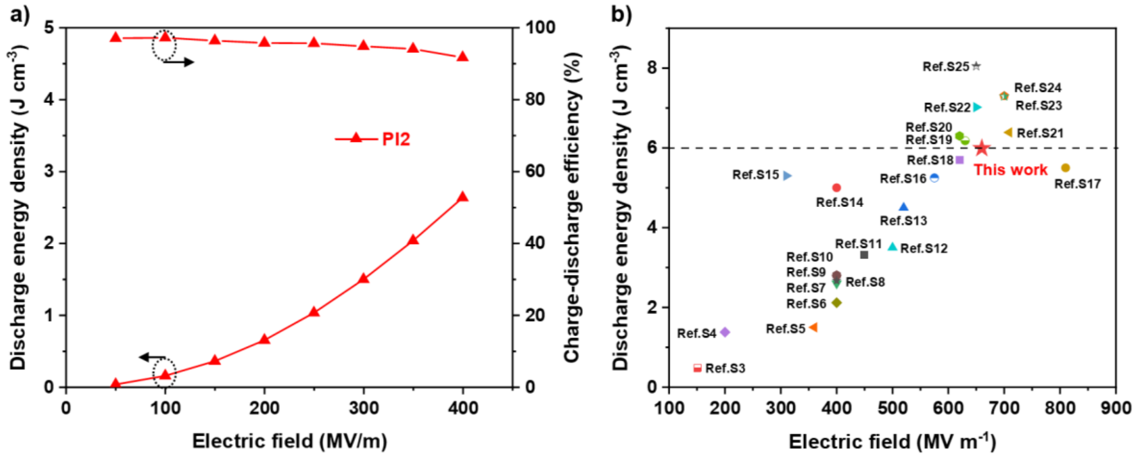

Figure S23. (a) Discharges energy density and charge-discharge efficiency of PI2 film at 200 ℃. (b) Comparisons of the discharged energy density of other references with the synthetic PI2 film at 150 ℃ (η > 90%).

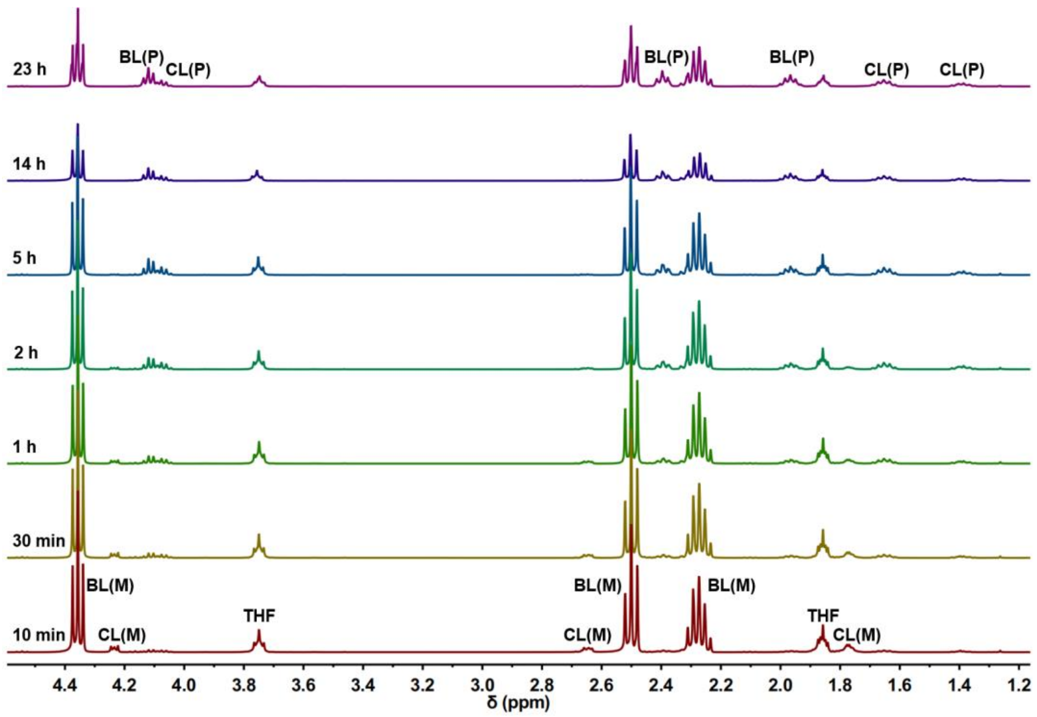

Figure S24. Overlay of ^{1}H-NMR ($CDCl_3$) spectra of copolymers P(BL-*co*-CL) quenched at different polymerization times. Reaction conditions: **Y-N** = 6.8 μmol, BnOH = 6.8 μmol, BL/CL/Y-N/BnOH = 2000/200/1/1, BL = 10.0 M in THF, copolymerize at -30 ℃.

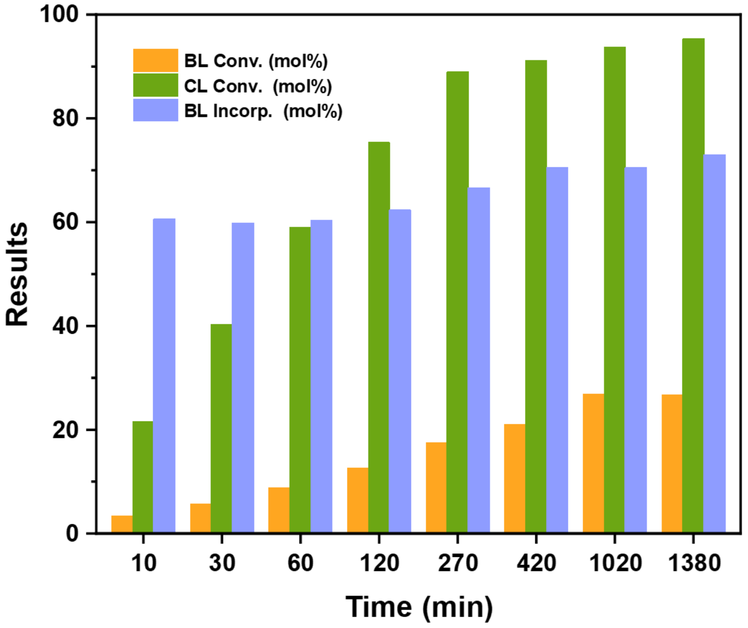

Figure S25. Time-dependent conversion of monomers (BL and CL) and BL incorporation during the copolymerization process.

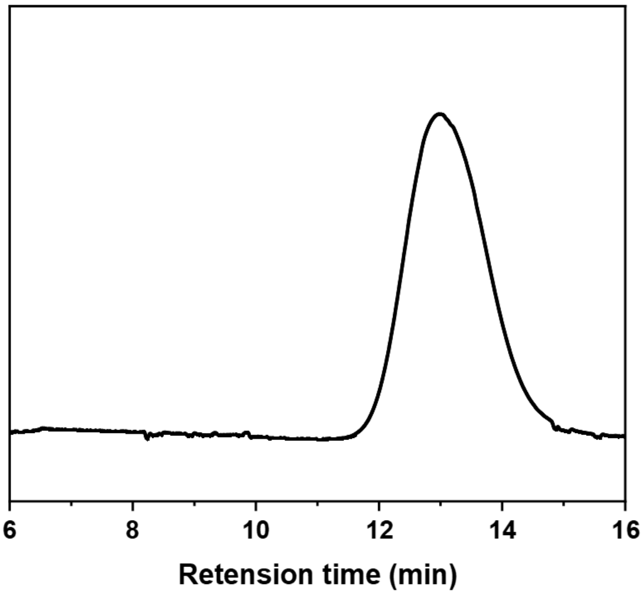

Figure S26. SEC trace of copolymer P(BL-*co*-CL) (M_n = 56.3 kg mol^{-1}, $Đ$ = 1.41).

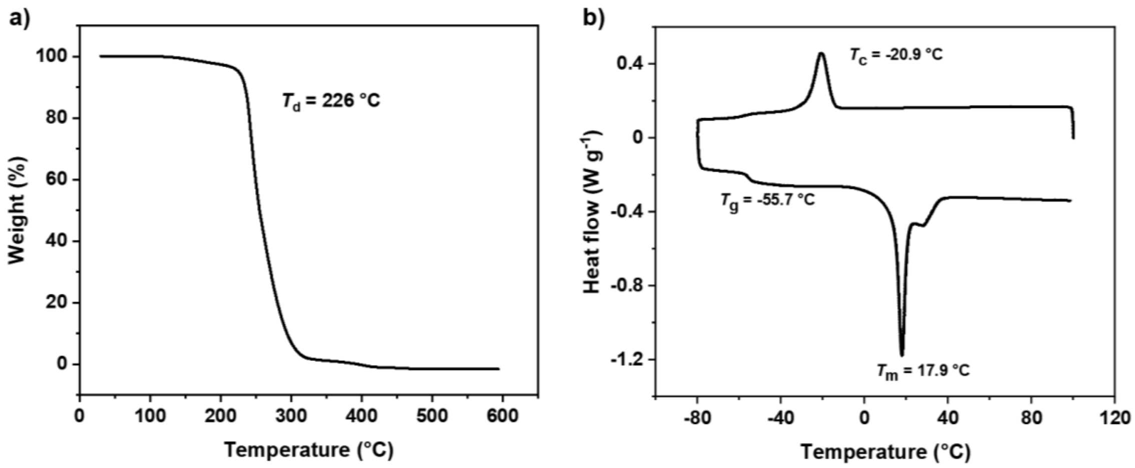

Figure S27. TGA (a) and DSC (b) curves of synthetic P(BL-*co*-CL).

Table S1. Products analysis of the hydrogenative depolymerization of different PU plastics[a].

Entry	PU plastics	Product Yield (%)	Products (mg)		
			Amines	Polyols	Lactones
1	Shoe sole	77	84	115	109
2	Tube	80	90	157	74
3	Tyre	86	164	137	44
4	Safety strip	82	158	88	82

[a]Reaction conditions: 400 mg PU plastic, 200 mg ZnO-ZrO_2/Cu, and 30 mL THF were stirred in an autoclave with 3 MPa (CO_2/H_2 = 1/3, v/v) at 200 ℃.

Table 2. Products analysis of the catalytic dehydrogenation of obtained BDO from waste PU tyre[a].

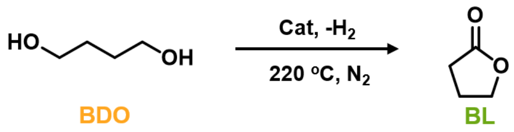

Entry	Time (h)	Con.$_{BDO}$ (%)	Liquid products (mmol)		Gas products (mmol)		
			BL	Byproduct	H_2	CO_2	CO
1	3	82	8.6	0.06	n.c.		
2	6	90	9.5	0.08	n.c.		
3	9	96	10.0	0.10	n.c.		
4	12	97	10.1	0.11	12.7	0.01	<0.01

[a]Reaction conditions: 0.95 g BDO (10.6 mmol), 200 mg ZnO-ZrO_2/Cu, and 40 mL THF were stirred in an autoclave with 1 MPa N_2 at 200 °C. Byproduct in the reaction is butyric anhydride, n.c. = not checked.

Table S3. Curve-fit Parameters [a] for Cu *K*-edge EXAFS of ZnO-ZrO_2/Cu.

Catalyst	Path	*d* (Å) [b]	C. N. [c]	ΔE_0 (eV)	σ^2 ($Å^2$) [d]	*R*-factor
Fresh	Cu-O	-	-	5 ± 1	-	0.007
	Cu-Cu	2.54 ± 0.01	9.7 ± 0.7		0.009 ± 0.001	
Spent	Cu-O	-	-	5 ± 1	-	0.003
	Cu-Cu	2.54 ± 0.01	10.8 ± 0.5		0.009 ± 0.001	

a: The data ranges used in these fittings are $3.0 \leq k \leq 12.0$ $Å^{-1}$ and $1.0 \leq R \leq 3.0$ Å. S_0^2 was fixed at 0.847, obtained from the Cu foil measured at the same time. The number of variable parameters is 4, out of total of 11.18 independent data point. *b*: The coordination distance. The distance for Cu-Cu is from the crystal structure of Cu (*Fm*-3*m*, ICSD collection code 136042). *c*: Average coordination number. *d*: Debye-Waller factor.

SI References

1. Hultzsch, K. C.; Voth, P.; Beckerle, K.; Spaniol, T. P.; Okuda, J. Single-component polymerization catalysts for ethylene and styrene: synthesis, characterization, and reactivity of alkyl and hydrido yttrium complexes containing a linked amido-cyclopentadienyl ligand. *Organometallics* **2000**, 19 (3), 93-101.
2. Amgoune, A.; Thomas, C. M.; Roisnel, T.; Carpentier, J.-F. Ring-opening polymerization of lactide with group 3 metal complexes supported by dianionic alkoxy-amino-bisphenolate ligands: combining high activity, productivity, and selectivity. *Chem. Eur. J.* **2006**, 12 (1), 169-179.
3. Zuo, P.; Li, J.; Chen, D.; Nie, L.; Gao, L.; Lin, J.; Zhuang, Q. Scalable co-cured polyimide/poly(*p*-phenylene benzobisoxazole) all-organic composites enabling improved energy storage density, low leakage current and long-term cycling stability. *Mater. Horiz.* **2024**, doi: 10.1039/d3mh01479g.
4. Dai, Z.; Bao, Z.; Ding, S.; Liu, C.; Sun, H.; Wang, H.; Zhou, X.; Wang, Y.; Yin, Y.; Li, X.; Scalable polyimide-poly(amic acid) copolymer based nanocomposites for high-temperature capacitive energy storage. *Adv. Mater.* **2022**, 34, 2101976.
5. Wu, C.; Deshmukh, A. A.; Li, Z.; Chen, L.; Alamri, A.; Wang, Y.; Ramprasad, R.; Sotzing, G. A.; Cao, Y.; Flexible temperature-invariant polymer dielectrics with large bandgap. *Adv. Mater.* **2020**, 32, 2000499.
6. Zhou, Y.; Li, Q.; Dang, B.; Yang, Y.; Shao, T.; Li, H.; Hu, J.; Zeng, R.; He, J.; Wang, Q. A scalable, high-throughput, and environmentally benign approach to polymer dielectrics exhibiting significantly improved capacitive performance at high temperatures. *Adv. Mater.* **2018**, 30, 1805672.
7. Wu, C.; Deshmukh, A. A.; Yassin, O.; Zhou, J.; Alamri, A.; Vellek, J.; Shukla, S.; Sotzing, M.; Casalini, R.; Sotzing, G. A.; Cao, Y. Flexible cyclic-olefin with enhanced dipolar relaxation for harsh condition electrification. *Proc. Natl. Acad. Sci. U S A* **2021**, 118, e2115367118.
8. Li, H.; Gadinski, M. R.; Huang, Y.; Ren, L.; Zhou, Y.; Ai, D.; Han, Z.; Yao, B.; Wang, Q. Crosslinked fluoropolymers exhibiting superior high-temperature energy density and charge-discharge efficiency. *Energy Environ Sci.* **2020**, 13, 1279-1286.
9. Dong, J.; Hu, R.; Xu, X.; Chen, J.; Niu, Y.; Wang, F.; Hao, J.; Wu, K.; Wang, Q.; Wang, H. A facile in situ surface-functionalization approach to scalable laminated high-temperature polymer dielectrics with ultrahigh capacitive performance. *Adv. Funct. Mater.* **2021**, 31, 2102644.
10. Song, J.; Qin, H.; Qin, S.; Liu, M.; Zhang, S.; Chen, J.; Zhang, Y.; Wang, S.; Li, Q.; Dong, L.; Xiong, C.; Alicyclic polyimides with large band gaps exhibit superior high-temperature capacitive energy storage. *Mater. Horiz.* **2023**, 10, 2139-2148.
11. Li, H.; Ai, D.; Ren, L.; Yao, B.; Han, Z.; Shen, Z.; Wang, J.; Chen, L.-Q.; Wang, Q. Scalable polymer nanocomposites with record high-temperature capacitive performance enabled by rationally designed nanostructured inorganic fillers. *Adv. Mater.* **2019**, 31, 1900875.

12. Cheng, S.; Zhou, Y.; Li, Y.; Yuan, C.; Yang, M.; Fu, J.; Hu, J.; He, J.; Li, Q. Polymer dielectrics sandwiched by medium-dielectric-constant nanoscale deposition layers for high-temperature capacitive energy storage. *Energy Storage Mater.* **2021**, 42, 445-453.
13. Yuan, C.; Zhou, Y.; Zhu, Y.; Liang, J.; Wang, S.; Peng, S.; Li, Y.; Cheng, S.; Yang, M.; Hu, J.; Zhang, B.; Zeng, R.; He, J.; Li, Q. Polymer/molecular semiconductor all-organic composites for high-temperature dielectric energy storage. *Nat. Commun.* **2020**, 11, 3919.
14. Zhang, T.; Chen, X.; Thakur, Y.; Lu, B.; Zhang, Q.; Runt, J.; Zhang, Q. M. A high scalable dielectric metamaterial with superior capacitor performance over a broad temperature. *Sci. Adv.* **2020**, 6, eaax6622.
15. Dong, J.; Li, L.; Qiu, P.; Pan, Y.; Niu, Y.; Sun, L.; Pan, Z.; Liu, Y.; Tan, L.; Xu, X.; Xu, C.; Luo, G.; Wang, Q.; Wang, H. Scalable polyimide-organosilicate hybrid films for high-temperature capacitive energy storage. *Adv. Mater.* **2023**, 35, 2211487.
16. Wang, P.; Yao, L.; Pan, Z.; Shi, S.; Yu, J.; Zhou, Y.; Liu, Y.; Liu, J.; Chi, J.; Wang, Q. Ultrahigh energy storage performance of layered polymer nanocomposites over a broad temperature range. *Adv. Mater.* **2021**, 33, 2103338.
17. Pei, Z.; Liu, Y.; Zhao, W.; Yang, C.; Li, S.; Jiang, P.; Chen, J.; Huang, X. Wide bandgap heterostructured dielectric polymers by rapid photo-crosslinking for high-temperature capacitive energy storage. *Adv. Funct. Mater.* **2023**, 2307639.
18. Yang, M.; Wang, Z.; Zhao, Y.; Liu, Z.; Pang, H.; Dang, Z.-M. Unifying and suppressing conduction losses of polymer dielectrics for superior high-temperature capacitive energy storage. *Adv. Mater.* **2023**, 2309640.
19. Chen, J.; Zhou, Y.; Huang, X.; Yu, C.; Han, D.; Wang, A.; Zhu, Y.; Shi, K.; Kang, Q.; Li, P. Jiang, P.; Qian, X.; Bao, Hua., Li, S.; Wu, G.; Zhu, X.; Wang, Q. Ladderphane copolymers for high-temperature capacitive energy storage. *Nature* **2023**, 615, 62-66.
20. Sun, B.; Hu, P.; Ji, X.; Fan, M.; Zhou, L.; Guo, M.; He, S.; Shen, Y. Excellent stability in polyetherimide/SiO_2 nanocomposites with ultrahigh energy density and discharge efficiency at high temperature. *Small*, **2022**, 18, 2202421.
21. Ren, W.; Yang, M.; Zhou, L. Fan, Y.; He, S.; Pan, J.; Tang, T.; Xiao, Y.; Nan, C.-W.; Shen, Y.; Scalable ultrathin all-organic polymer dielectric films for high-temperature capacitive energy storage. *Adv. Mater.* **2022**, 34, 2207421.
22. Pan, Z.; Li, L.; Wang, L.; Luo, G.; Xu, X.; Jin, F.; Dong, J.; Niu, Y.; Sun, L.; Guo, C.; Zhang, W.; Wang, Q.; Wang, H. Tailoring poly(styrene-co-maleic anhydride) networks for all-polymer dielectrics exhibiting ultrahigh energy density and charge-discharge efficiency at elevated temperatures. *Adv. Mater.* **2023**, 35, 2207580.
23. Ran, Z.; Wang, R.; Fu, J.; Yang, M.; Li, M.; Hu, J.; He, J.; Li, Q. Spiral-structured dielectric polymers exhibiting ultrahigh energy density and charge-discharge efficiency at high temperatures. *Adv. Mater.* **2023**, 2303849.
24. Wang, R.; Zhu, Y.; Fu, J.; Yang, M.; Ran, Z.; Li, J.; Li, M.; Hu, J.; He, J.; Li, Q. Designing tailored combinations of structural units in polymer dielectrics for

high-temperature capacitive energy storage. Designing tailored combinations of structural units in polymer dielectrics for high-temperature capacitive energy storage. *Nat. Commun.* **2023**, 14, 2406.
25. Yang, M.; Zhou, L.; Li, X.; Ren, W.; Shen, Y. Polyimides physically crosslinked by aromatic molecules exhibit ultrahigh energy density at 200 ℃. *Adv. Mater.* **2023**, 35, 2302392.
